# Modulating versatile pathways using a cleavable PEG shell and EGFR-targeted nanoparticles to deliver CRISPR-Cas9 and docetaxel for triple-negative breast cancer inhibition

**DOI:** 10.1007/s12272-024-01514-0

**Published:** 2024-11-01

**Authors:** Yu-Li Lo, Ci-Jheng Hong, Chen-Shen Wang, Ching-Ping Yang

**Affiliations:** 1 Institute of Pharmacology, National Yang Ming Chiao Tung University, Taipei, 112 Taiwan; 2 Faculty of Pharmacy, National Yang Ming Chiao Tung University, Taipei, 112 Taiwan; 3 Department of Pharmacy, Antai Medical Care Corporation Antai Tian-Sheng Memorial Hospital, Pingtung, Taiwan

**Keywords:** HuR knockout by CRISPR/Cas9, Chemotherapy, pH-sensitive targeting nanoparticle, Epidermal growth factor receptor (EGFR), Progression, Triple-negative breast cancer

## Abstract

Human antigen R (HuR), an RNA-binding protein, is implicated in regulating mRNA stability and translation in cancer, especially in triple-negative breast cancer (TNBC), a highly aggressive form. CRISPR/Cas9-mediated HuR knockout (HuR CRISPR) presents a promising genetic therapeutic approach, but it encounters transfection limitations. Docetaxel (DTX), an effective cytotoxic agent against metastatic breast cancer (BC), faces challenges related to vehicle-associated adverse events in DTX formulations. Therefore, we designed multifunctional nanoparticles with pH-sensitive PEG derivatives and targeting peptides to enable efficient HuR CRISPR and DTX delivery to human TNBC MDA-MB-231 cells and tumor-bearing mice. Our findings indicated that these nanoparticles displayed pH-responsive cytotoxicity, precise EGFR targeting, efficient tumor penetration, successful endosomal escape, and accurate nuclear and cytoplasmic localization. They also demonstrated the ability to spare normal cells and prevent hemolysis. Our study concurrently modulated multiple pathways, including EGFR, Wnt/β-catenin, MDR, and EMT, through the regulation of EGFR/PI3K/AKT, HuR/galectin-3/GSK-3β/β-catenin, and P-gp/MRPs/BCRP, as well as YAP1/TGF-β/ZEB1/Slug/MMPs. The combined treatment arrested the cell cycle at the G2 phase and inhibited EMT, effectively impeding tumor progression. Tissue distribution, biochemical assays, and histological staining revealed the enhanced safety profile of pH-responsive PEG- and peptide-modified nanoformulations in TNBC mice. The DTX-embedded and peptide-modified nanoparticles mitigated the side effects of DTX, enhanced cytotoxicity in TNBC MDA-MB-231 cells, and exhibited remarkable antitumor efficacy and safety in TNBC-bearing mice with HuR CRISPR deletion. Collectively, the combination therapy of DTX and CRISPR/Cas9 offers an effective platform for delivering antineoplastic agents and gene-editing systems to combat tumor resistance and progression in TNBC.

**Supplementary Information:**

The online version contains supplementary material available at 10.1007/s12272-024-01514-0.

## Introduction

Breast cancer (BC) is one of the most widespread and invasive cancer types among women worldwide (Azadnajafabad et al. 2023). Triple-negative breast cancer (TNBC), comprising 10–20% of all breast cancers, exhibits heightened aggressiveness, poor prognosis, and shorter survival rates than other BC subtypes (Karn et al. 2022). Although chemotherapy is one of the major regimens used to treat TNBC, the success of chemotherapeutic agents is still limited due to the lack of well-defined molecular targets (Hossein-Nejad-Ariani et al. 2019; Karn et al. 2022). EGFR upregulation is frequently noticed in numerous TNBC cells, and thus EGFR is a potential delivery target for TNBC treatment (Subham et al. 2022). In the present study, human MDA-MB-231 cells displaying high aggression, metastasis, poor differentiation, and EGFR overexpression were selected as a model of TNBC (Wendt et al. 2015).

Docetaxel (DTX) remains a cornerstone in current chemotherapeutic strategies for TNBC treatment (Chaurawal and Raza 2023). This cytotoxic taxane and antimicrotubular agent, derived from the European Yew Taxus baccata, effectively modulates microtubule polymerization and arrests the cell cycle in the M phase, contributing to cell death (van Eijk et al. 2022). Approved for use as a single agent or in combination therapy, DTX is utilized in the treatment of BC, gastric cancer (GC), hormone-refractory prostate cancer, and various tumor types (Rijcken et al. 2022; Chaurawal and Raza 2023). Despite being effective, the utility of DTX is hampered by drug-associated adverse events and toxicities, including neutropenia and vomiting (Gupta et al. 2022). A notable concern in commercial DTX formulations is Cremophor EL, a vehicle aiding DTX dissolution (Markman 2003; Rijcken et al. 2022). Additionally, epithelial-to-mesenchymal transition (EMT) and chemoresistance are crucial challenges for treating TNBC, which may account for DTX treatment failure and high recurrence (Nozaki et al. 2023). DTX is a substrate of P-glycoprotein (P-gp), leading to DTX efflux and multidrug resistance (MDR) of BC cells to chemotherapeutics (Seneme et al. 2022).

Moreover, there is a growing recognition of the role of post-transcriptional regulation by human antigen R (HuR), encoded by ELAVL1, in modulating signaling pathways involved in proliferation, angiogenesis, progression, and MDR (Wang et al. 2021; Yang et al. 2021). HuR upregulation, linked to aggressive malignancy and poor prognosis, is observed in several tumor types, including BC, such as the MDA-MB-231 cell line (Liao et al. 2023). Functioning by binding to adenylate-uridylate (AU)-rich elements in the 3’ untranslated region of RNA transcripts, such as Bcl-2 and β-catenin, HuR regulates oncogenes, including c-Myc and cyclin D1 (Lin et al. 2017; Pascale et al. 2022). Inhibition of HuR in MDA-MB-231 cells is anticipated to enhance DTX therapeutic effects and potentiate BC cell death. Advancing this understanding, our study employs the clustered regularly interspaced short palindromic repeats (CRISPR)/Cas9 system, specifically **HuR CRISPR**, as an advanced gene-editing tool (Wang et al. 2021). This approach involves a single guide RNA (sgRNA) that guides the Cas9 nuclease to cleave the targeted HuR DNA, inducing double-stranded breaks (DSBs) at specific genomic loci to diminish HuR’s functions in BC progression.

To ensure the integrity of DTX and HuR CRISPR, we engineered versatile nanoparticles coated with multifunctional peptides and acidic tumor microenvironment (TME)-sensitive peptides, thereby establishing a promising delivery system for the combined therapy of DTX and HuR CRISPR. Solid lipid nanoparticles (SLN) and liposomes (Lip), vanguard nanocarriers in cancer treatment, are emerging as potent modalities for addressing critical challenges in both pre-clinical and clinical settings (Wang et al. 2021; Lo et al. 2022; Tran et al. 2023; Li et al. 2024). The remarkable efficacy of SLN facilitates the delivery of nucleic acids such as HuR CRISPR into cancer cells (Wang et al. 2021). Furthermore, liposomes demonstrate exceptional capability in transporting lipophilic drugs such as DTX (Tran et al. 2023). These advancements in nanocarrier technology present potential solutions to the challenges faced by current therapies, encompassing issues, including the rapid degradation of nucleic acids and the restricted penetration and uptake of therapeutics into tumor cells (Lo et al. 2022).

The incorporation of cell-penetrating peptides (CPPs), consisting of 5–30 cationic amino acids, onto these nanoparticles enhances cellular uptake, specific tumor targeting, and pH sensitivity (Wang et al. 2021; Lo et al. 2022). Notably, the **H peptide**, characterized by pH sensitivity, responds to the acidic pH prevalent in the TME, endosomes, and lysosomes (Wang et al. 2021). Due to its high histidine content, the imidazole ring on the histidine of H peptide undergoes protonation under low pH conditions, resulting in increased hydrophilicity (Ju et al. 2017). **A peptide**, a neutral-charged EGFR targeting ligand (Han et al. 2013), aligns perfectly with the high EGFR expression levels observed in MDA-MB-231 cells (Wischmann et al. 2023). Consequently, A peptide emerges as a functionally specific target, binding selectively to EGFR in BC cells. Furthermore, **R peptide**, a cationic CPP derived from human phosphatidate phosphatase, directs cargoes to the cell nucleus (Lo et al. 2022; Geng et al. 2023). This intricate design harnesses the strengths of various peptides, optimizing their functionalities for a robust and targeted therapeutic approach.

**Fig. 1:**
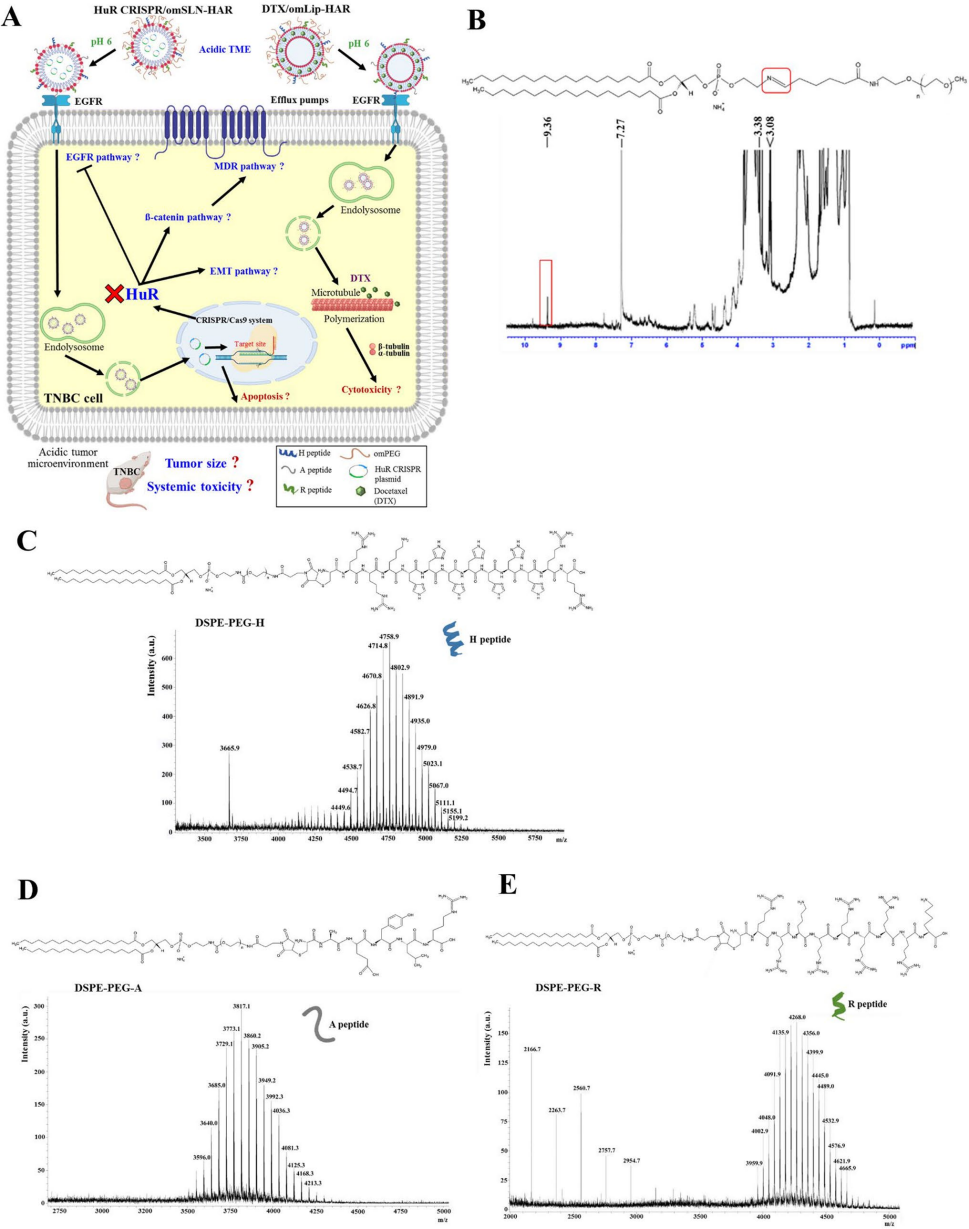
**A** pH-sensitive and targeted nanoparticles for TNBC therapy. The diagram illustrates the design of HuR CRISPR/omSLN-HAR and DTX/omLip-HAR nanoparticles, showcasing their potential for regulating signaling pathways and suppressing tumors in the acidic tumor microenvironment (TME) of TNBC. (B) ^1^H NMR of DSPE-imine-omPEG conjugation. **C**–**E** Structure and spectra of DSPE-PEG-peptides. Mass spectra of the lipid-peptide conjugates, specifically **C** DSPE-PEG-H, **D** DSPE-PEG-A, and **E** DSPE-PEG-R, were obtained using MAL-DI-TOF mass spectrometry

The lipid shells of SLN and Lip were adorned with H, A, and R peptides to create SLN-HAR and Lip-HAR. This modification aimed to achieve pH sensitivity, EGFR targeting, tumor penetration, endosomal escape, and localization to the nucleus and cytoplasm. In addition, the PEG derivative Oʹ-methyl polyethylene glycol (omPEG) was conjugated with lipids, forming a pH-sensitive imine bond. SLN-HAR and Lip-HAR were further coated with this degradable long-chain omPEG to yield omSLN-HAR and omLip-HAR. This dual-nanoparticle design, incorporating distinct peptides and an acidic TME-sensitive PEG derivative, not only mitigates the toxic effects on normal cells but also enhances the specific ligand-receptor binding and uptake of nanoparticles into BC cells, facilitating the release of HuR CRISPR and DTX in the nucleus and cytoplasm. A schematic representation of the HuR CRISPR/omSLN-HAR and DTX/omLip-HAR designs is shown in Fig. 1A.

## Materials and methods

### Materials

The HuR CRISPR plasmid and surrogate plasmid were customized by Dr. Tseng Tsai-Yu, Cancer Progression Research Center, National Yang Ming Chiao Tung University (Taipei, Taiwan). Docetaxel was bought from MedChem Express (Monmouth Junction, NJ, USA). H, A, and R peptides were synthesized by Kelowna in Taipei, Taiwan. Cholesterol and paraformaldehyde were purchased from Acros (Geel, Belgium). All lipids were purchased from Avanti (Alabaster, AL, USA). All cell culture media and reagents were purchased from Gibco BRL (Grand Island, NY, USA). The majority of other chemical compounds and reagents were procured through MedChem Express, Cayman (Ann Arbor, MI, USA), or Merck KGaA (Darmstadt, Germany), including Millipore Sigma (St. Louis, MO, USA).

### Synthesis of the peptide-conjugated lipid

DSPE-PEG-maleimide and peptides H, A, and R were dissolved in methanol at a 1:1 molar ratio. After a 24-h reaction in the dark at room temperature, the mixture was evaporated, and the residue was re-dissolved in water. The solution was then dialyzed overnight against water using a dialysis bag (1000–3500 MWCO). The final product (DSPE-PEG-peptide) was lyophilized, and the molecular weight of peptide-conjugated lipid was confirmed using matrix-assisted laser desorption ionization time-of-flight mass spectrometry (MALDI-TOF MS; Bruker, Billerica, MA, USA).

### Synthesis of DSPE–omPEG

DSPE and omPEG were dissolved in chloroform and methanol for 24 h. The mole ratio of DSPE to omPEG was 1:1. The organic solvent was removed using an evaporator to prepare DSPE-omPEG. Successful conjugation of the imine bond in DSPE-omPEG was confirmed using ^1^H NMR (400 MHz, Bruker Avance III, Bruker UK Ltd., Coventry, UK).

### Preparation of pH-sensitive, peptide-conjugated, and HuR CRISPR-loaded SLN formulations

L-α-Phosphatidylcholine (PC), cholesterol, DOTAP, DSPE-PEG-peptide, and DSPE-omPEG were dissolved in ethanol at a molar ratio of 1:0.1:0.1:0.1:0.1 while heating and stirring at 50 °C. Tween 80 was added dropwise to the mixture and stirred for 1 h. After cooling to 37 °C, the CRISPR/Cas9 plasmid was gently added to the SLN dispersion to construct HuR CRISPR/omSLN-HAR.

### Preparation of pH-sensitive, peptide-conjugated, and DTX-embedded Lip formulations

DTX-embedded liposomes were prepared using the thin film hydration method. DSPC, cholesterol, DSPE-PEG-peptide, DTX, and DSPE-omPEG were dissolved in a round-bottomed flask. After evaporating the organic solvent using a rotary evaporator at 55 °C, the lipid thin film was resuspended in PBS by water bath sonication. The liposome mixture was extruded through 200- and 100-nm membrane filters 4 times.

### Characterization of omSLN-HAR and omLip-HAR

The particle size and zeta potential of HuR CRISPR/omSLN-HAR and DTX/omLip-HAR were measured using a Zetasizer Nano-ZS particle size analyzer from Malvern (Worcestershire, England, UK). The morphology of the prepared nanoparticles was observed by transmission electron microscopy (TEM; JEM-2000EXII, Tokyo, Japan). Nanoparticles were fixed on carbon-coated copper grids and negatively stained with 2% uranyl acetate.

### Stability of the SLN and Lip

To assess the stability of various SLN and Lip formulations, we monitored changes in particle size, polydispersity index (PDI), and zeta potential over 24 weeks at 4 °C. The measurements followed the same procedure as previously mentioned.

### Encapsulation efficiency (EE%) and drug loading efficiency (DL%)

The HuR CRISPR- and DTX-containing nanoparticle dispersion was subjected to centrifugation at 15,000 rpm for 15 min to separate the free HuR CRISPR plasmid and DTX. HuR CRISPR was analyzed using NanoDrop. DTX was analyzed using an HPLC system, which included an L7100 pump, 1210 Primaide autosampler, LiChrospher column, and L2400 UV detector at a wavelength of 229 nm. Each sample was detected in triplicate. The EE% or DL% of DTX or HuR CRISPR in Lip and SLN were then calculated using the following formula.

1 $$ {\text{EE}}\% \, = \, \left[ {\left( {{\text{W}}_{{\text{e}}} {-}{\text{ W}}_{{\text{f}}} } \right){\text{/W}}_{{\text{e}}} } \right] \times 100\% $$

2 $$ {\text{DL}}\% \, = \, \left[ {\left( {{\text{W}}_{{\text{e}}} {-}{\text{ W}}_{{\text{f}}} } \right){\text{/W}}_{{\text{t}}} } \right] \times 100\% $$

W_e_ is the weight of added DTX or HuR CRISPR, W_f_ is the weight of DTX or HuR CRISPR in the supernatant, and W_t_ is the total weight of the nanoparticle.

### Drug release triggered by pH-sensitivity

A volume of one milliliter of nanoparticle dispersions was encapsulated within a dialysis bag with a molecular weight cutoff range of 1000–3500. The nanoparticles were then placed in 30 ml of release media at pH 7.4 or 6.0. The respective formulations were incubated at 37 °C for specified time intervals (0, 1, 2, 4, 8, 12, 24, 48, and 72 h). HuR CRISPR and DTX concentrations were determined using NanoDrop and HPLC, separately.

### Cell lines

MDA-MB-231 cells represent human tongue squamous cell carcinoma and were maintained in Dulbecco’s Modified Eagle’s Medium (DMEM) supplemented with 10% fetal bovine serum (FBS) and 1% penicillin–streptomycin solution (P/S). Normal mouse embryonic fibroblasts (NIH/3T3 cells) were cultured in DMEM supplemented with 10% bovine calf serum (BCS) and 1% P/S.

### Cellular uptake

The cellular uptake of MDA-MB-231 and NIH/3T3 cells was quantified using FACSCalibur™ flow cytometry (BD Biosciences, San Jose, CA, USA). After treatment and centrifugation, cell pellets were collected and resuspended in 1 mL of PBS after 24-h incubation with GFP-plasmid or DiI-DTX in various formulations. The fluorescence intensity of the GFP-plasmid or DiI-DTX internalized by the cells was assessed via flow cytometry.

### Identification of intracellular localization

MDA-MB-231 cells were seeded in 6-well plates with 2 ml of medium. After incubation with GFP-plasmid in SLN formulations and DiI-DTX in Lip formulations, the cells were fixed in 4% paraformaldehyde, and LysoTracker® Red/Green was added to monitor lysosome localization. The primary antibodies against EGFR or alpha-tubulin and the secondary antibodies against Cy5.5 were used for immunofluorescence staining. Cells were stained with DAPI at room temperature to observe the nucleus. Images were taken using a confocal laser scanning microscope (CLSM; Olympus FV10i, Tokyo, Japan).

### Design of the HuR sgRNA

The sequence of the HuR (ELAVL1) gene was obtained from the human genome database at NCBI. Exon 1 of HuR was analyzed using the online CRISPR sgRNA Design Tool. The tool provided information on the protospacer adjacent motif (PAM) site and off-target efficiency and specificity scores. We carefully chose two sgRNA sequences characterized by enhanced targeting specificity and reduced off-target effects and employed these sequences for subsequent experiments.

### Identification of HuR CRISPR cells

A surrogate plasmid serves as a reporter system designed to interact with the target sequence. These plasmids contain genes that encode for GFP (green fluorescent protein) and tdTOMATO (red fluorescent protein), connected by the HuR targeting sequence. When the HuR protein is expressed, cells emit green fluorescence due to the GFP gene because tdTOMATO is in-frame. However, if the HuR sequence is knocked out by the CRISPR/Cas9 system, this results in frameshift mutations, leading to the expression of the red fluorescent protein, indicating the absence of the HuR sequence in the cells. MDA-MB-231 cells were treated with a HuR CRISPR plasmid and a surrogate plasmid encapsulated in SLN formulations or commercial transfection agents (for comparison) at 37 °C for 24 h. After confirming the fluorescence signals using a Nikon fluorescence microscope, cells exhibiting both green and red fluorescence were sorted using a FACSAria cell sorter (BD, San Jose, CA, USA).

### Transfection and HuR knockout efficiency analysis

Quantification of transfection efficiency in 3T3/NIH and MDA-MB-231 cells was assessed using flow cytometry. Following incubation of cells with GFP-plasmid using SLN formulations or commercial transfection agents at 37 °C for 24 h, cell pellets were collected and resuspended in PBS. The transfection efficiency and HuR knockout efficiency induced by the CRISPR/Cas9 system in the cells were measured using a FACSCalibur™ flow cytometer.

### Identification of the HuR sequence in MDA-MB-231 cells with and without HuR CRISPR modification

DNA from MDA-MB-231 cells, both with and without HuR CRISPR modification, was extracted using the gSYNC™ DNA Extraction Kit (Geneaid Biotech Ltd., New Taipei City, Taiwan). The HuR gene sequences in the extracted DNA were then amplified by polymerase chain reaction (PCR). The amplified HuR DNA sequences underwent polyacrylamide gel electrophoresis (PAGE) with 0.001% ethidium bromide. The gel results were visualized using the DigiGel Gel Documentation System. Subsequently, the amplified DNA sequences from MDA-MB-231 cells, both with and without HuR CRISPR knockout, were sequenced using primers and the BigDye Terminator v3.1 Cycle Sequencing Kit (Applied Biosystems, MA, USA). The entire sequencing reaction was loaded onto a DNA Analyzer (Applied Biosystems), and the sequencing data were analyzed using Chromas v1.0.

### Cell viability assessment using the sulforhodamine B (SRB) assay

The SRB assay was employed to evaluate cell viability. In 96-well plates, NIH/3T3 and MDA-MB-231 cells were seeded. These cells were subjected to treatment with varying concentrations of DTX, DTX-Lip, or DTX-Lip-HAR with and without HuR CRISPR modification, and were then maintained at 37 °C for 48 h. Subsequently, a 0.04% SRB solution was introduced, allowing for a 30-min incubation. Each well underwent three washes with 1% acetic acid. After overnight air-drying at room temperature, 10 mM Tris-base was added to each well. The absorbance was measured at a wavelength of 540 nm using an ELISA reader (TECAN Sunrise, Männedorf, Switzerland).

### Cell cycle analysis

To determine the distribution of cells in the G0/G1, S, and G2/M phases of the cell cycle, propidium iodide (PI) DNA staining was employed. Specifically, MDA-MB-231 cells were seeded in 24-well plates and incubated overnight. Subsequently, the cells were treated with various formulations of DTX (0.04 μM, IC30) and incubated at 37 °C for 48 h. The cells were collected and fixed in 70% ethanol in PBS at − 20 °C overnight. After removal of ethanol/PBS through centrifugation, the cells were incubated with PI solution for 30 min in the dark at room temperature. The collected cells were subsequently washed with PBS and analyzed using a FACSCalibur™ flow cytometer.

### Annexin V/PI staining

MDA-MB-231 cells, both with and without HuR CRISPR/SLN-HAR modification, were seeded in 24-well plates. Various formulations of DTX (0.04 μM, IC30) were added and incubated at 37 °C for 48 h. Cells were collected and stained with Annexin V-FITC/PI double staining detection kit (Strong Biotech Corporation, Taiwan) in the dark at room temperature. The collected cells were detected using a FACSCalibur flow cytometer. Phosphatidylserine is exposed in early apoptotic cells with intact cell membranes, binding to Annexin V-FITC, and appearing in the Annexin V^+^/PI^−^ quadrant. As cells progress into necrosis or late apoptosis, they shift into the Annexin V^+^/PI^+^ quadrant. Distinct cell populations, including viable cells (Annexin V^−^/PI^−^), early apoptotic cells (Annexin V^+^/PI^−^), late apoptotic or necroptotic cells (Annexin V^+^/PI^+^), and dead cells (Annexin V^−^/PI^+^), were identified by flow cytometry.

### Western blotting assay

MDA-MB-231 cells, both with and without HuR CRISPR/SLN-HAR modification, were seeded in 6-cm dishes and incubated overnight. Various treatments were added, and the cells were cultured for 48 h. Cellular proteins were lysed using RIPA buffer, and their concentrations were measured using the BCA Protein Assay Kit (Thermo Fisher Scientific, Waltham, MA, USA). The protein samples were separated by sodium dodecyl sulfate (SDS)–PAGE and transferred onto polyvinylidene fluoride (PVDF) membranes. Following a 1-h blocking step with 5% non-fat milk, the blots were incubated at 4 °C overnight with primary antibodies against specific proteins. Subsequently, the blots were incubated with secondary antibodies at room temperature for 1 h. Relative protein expression levels were visualized and captured using a Luminescence Imaging System (Amersham™ Imager 680) with Pierce™ enhanced chemiluminescence (ECL) kits (Thermo Fisher Scientific, Waltham, MA, USA).

### Real-time PCR assay

After seeding, MDA-MB-231 cells with and without HuR CRISPR/SLN-HAR modification in 6-cm dishes overnight, the cells were incubated with various formulations for 48 h. Next, the cells were harvested and then treated with TRIzol reagent (Thermo Fisher Scientific, Waltham, MA, USA). Total RNA was extracted from chloroform and purified by isopropanol precipitation. RNA was transcribed into cDNA to determine gene expression at the mRNA level. The resulting cDNA was subjected to real-time PCR amplification using SYBR Green PCR Master Mix (Thermo Fisher Scientific, Waltham, MA, USA). Gene expression was normalized to GAPDH.

### Migration assay

MDA-MB-231 and HuR CRISPR MDA-MB-231 cells were seeded in culture inserts (Greiner, Frickenhausen, Germany) and incubated overnight. After this incubation period, the culture inserts were removed. The cells were subsequently treated with various formulations at 37 °C for 18 h. Images of cell migration were captured using optical microscopy. The migration area was quantified using ImageJ software. The relative migration percentage was calculated using the following equation:

3 $$ \text{Migration} \, \text{area} \, \left( {{\text{\% of the area before treatment}}} \right) = \, 100\% \, - \, \left[ {{\text{Blank area}}_{{({\text{after treatment}})}} /{\text{Blank area}}_{{({\text{before treatment}})}} \times \, 100\% } \right] $$

### MDA-MB-231 tumor-bearing mice with and without HuR CRISPR modification

Female BALB/c mice, approximately 6 weeks old with an average body weight of approximately 20 g, were procured from the National Laboratory Animal Center and housed at the National Yang Ming Chiao Tung University (NYCU) Animal Center. All procedures related to the care and handling of the animals were conducted in strict accordance with the guidelines approved by the Institutional Animal Care and Use Committee (IACUC) of NYCU. Female mice received a subcutaneous injection on the right side with a cell suspension of MDA-MB-231 or HuR CRISPR-modified MDA-MB-231 cells. The tumors were allowed to grow until they reached a size of approximately 100 mm^3^ before initiating the designated treatment. Tumor volume (V) was determined using the following formula:

4 $$ {\text{V }}\left( {{\text{mm}}^{{3}} } \right) \, = \, 0.5 \, \times {\text{ L }} \times {\text{ W}}^{2} $$

where L is the longest diameter (mm) and W is the shortest diameter (mm) perpendicular to the longest axis.

### Antitumor efficacy and body weight of tumor-bearing mice

Each mouse was subcutaneously administered 200 μl of one of the following formulations: PBS, DTX, DTX/Lip, DTX/Lip-HAR, and DTX/omLip-HAR with and without HuR CRISPR/SLN-HAR modification. In the DTX-containing groups, a dosage of 5 mg of DTX per kilogram of body weight was administered. These treatments, and the measurement of tumor size using a digital caliper, were performed every 3 days over 2 weeks. Tumor volume (V) was calculated using the formula mentioned earlier. In addition, the mice’s body weights were measured every 3 days using an electronic balance.

### Positron emission tomography/computed tomography (PET/CT)

Tumor images were monitored using PET/CT. On the last day of treatment, the tumor-bearing mice were anesthetized first and intravenously injected with [^18^F]-fluorodeoxyglucose (^18^F-FDG). After injection of ^18^F-FDG, images were obtained at 30 min using the Triumph pre‐clinical LabPET/X‐SPECT imaging system (TriFoil Imaging Inc., Northridge, CA, USA). The acquisition of CT images was performed for 3 min after each PET scan and detected anatomical diagrams of each animal using the MILabs uCT (MILabs, Houten, the Netherlands). The emission data were normalized and calibrated according to the tracer decay time. PET and CT images were observed and measured using AMIDE software (SourceForge, Iowa, USA).

### Biochemical tests, blood cell analysis, and a biodistribution study

After the final treatment, 200 μL blood samples were collected from the tumor-bearing mice. Plasma was obtained by centrifuging blood at 1000 rpm for 10 min at 4 °C. The levels of creatine kinase-MB (CK-MB), glutamic oxaloacetic transaminase (GOT), and blood urea nitrogen (BUN) were determined using a clinical dry chemistry analyzer (Fuji Dri-Chem 7000 V). Additionally, the levels of white blood cells (WBC), red blood cells (RBC), and platelets (PLT) were analyzed using a hematology analyzer (Sysmex XT-1800iv, Sysmex, Hyogo, Japan).

After sacrificing the mice, tissue samples were quickly frozen in liquid nitrogen and stored at − 80 °C. Approximately 100–200 mg of each tissue was placed in a glass vial. The organ samples were manually ground using a mortar and pestle. Methanol and chloroform were added for continued grinding, and the mixture was then transferred to a centrifuge tube. After vortexing, the samples were centrifuged at 3000 rpm at 4 °C for 30 min. The upper layer of each sample contained the extracted DTX. The amount of DTX in each tissue was estimated using the HPLC system, as previously described.

### Hematoxylin and eosin (H&E) staining and the terminal deoxynucleotidyl transferase dUTP nick end labeling (TUNEL) assay

The tumors, hearts, livers, kidneys, and intestines were fixed in 4% paraformaldehyde overnight, embedded in paraffin, and cut into 5 μm-thick sections for H&E staining. The histological images of these samples were taken up by using an Olympus microscope.

Additionally, the tumor samples were first frozen and then fixed in 4% paraformaldehyde for 20 min. Subsequently, the samples were washed with PBS and submerged in ice. Next, the samples were processed according to the manufacturer’s instructions using the In Situ Cell Death Detection Kit (Roche, Mannheim, Germany). Slices of the tumor samples were stained with Hoechst and visualized using an OLYMPUS FV10i CLSM.

### Statistical analysis

All experimental data are presented as mean ± standard deviation (SD) and analyzed using Student’s t-test. Statistical significance was indicated as follows: *p < 0.05, **p < 0.01, and *** *p* < 0.001.

## Results

### Physicochemical characterization of HuR CRISPR and DTX in different formulations

The ^1^H NMR spectrum of DSPE–omPEG displayed a peak at 9.36 ppm in Fig. 1B, indicating that a pH-sensitive imine bond was formed between DSPE and omPEG, as previously reported by our group (Juang et al. 2019). The mass spectra of DSPE-PEG-H, DSPE-PEG-A, and DSPE-PEG-R peptides were obtained via mass spectrometry, as shown in Fig. 1C–E, confirming the successful conjugation of the respective peptides to DSPE-PEG.

**Fig. 2:**
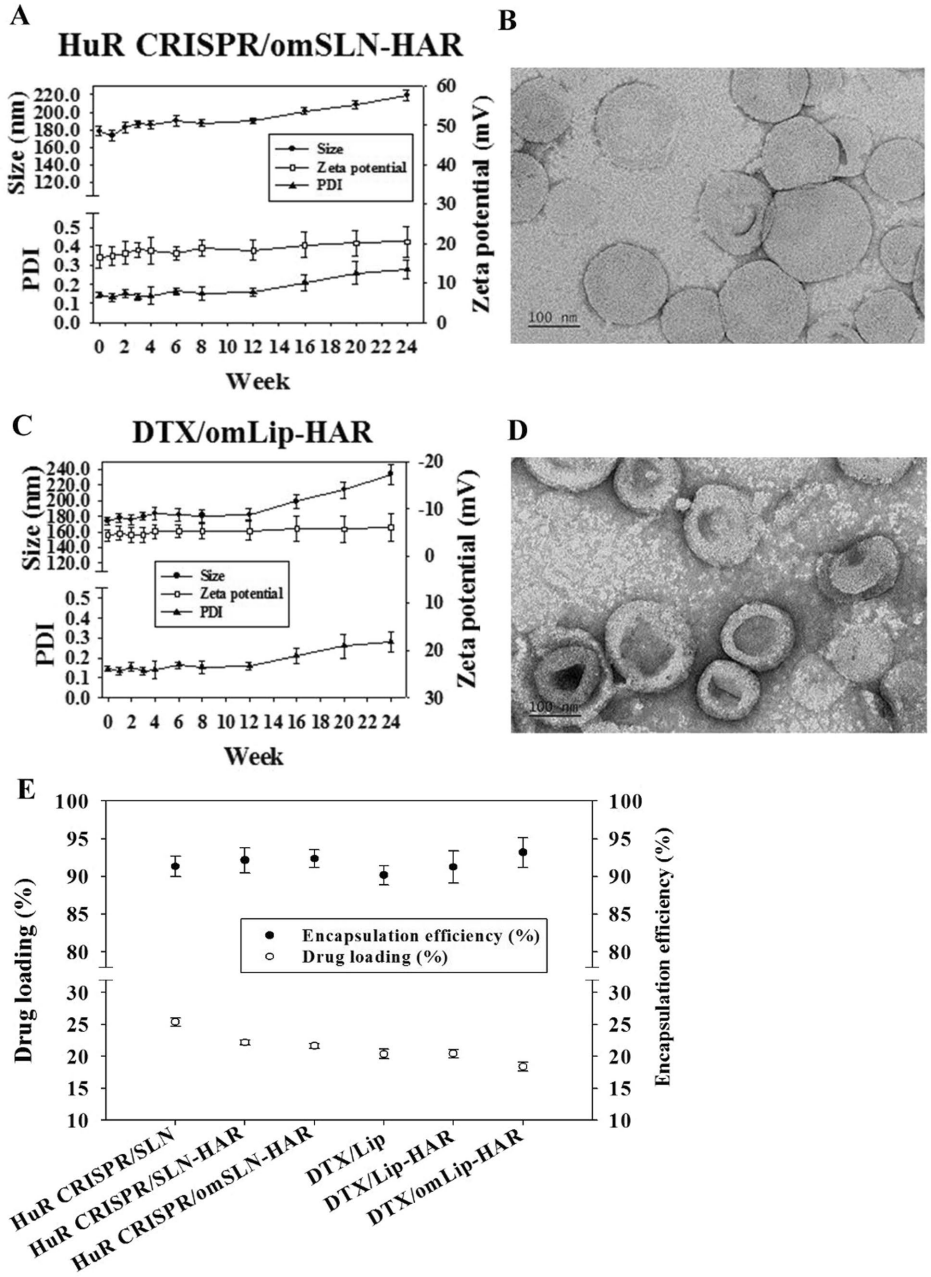
**A** Long-term stability and **B** TEM image of HuR CRISPR/omSLN-HAR. **F**–**H** Stability of DTX in Lip formulations; **C** Long-term stability and **D** TEM image of DTX/omLip-HAR. (**E**) Encapsulation efficiency % and drug loading % of various formulations. For each group, n = 3

The SLN and Lip preparations revealed particle dimensions in the nanoscale range (< 200 nm) (Table 1), whereas the dimensions and zeta potential of these nanoscale particles exhibited stability with a slender size distribution (PDI = 0.14 ~ 0.21) after storage at 4 °C for 24 weeks (Fig. 2A, C, S1A, B, D, E). This implies that the formulations underwent minimal alteration, thereby affirming their commendable long-term stability. HuR CRISPR/omSLN-HAR and DTX/omLip-HAR displayed spherical morphologies, as evidenced by TEM imaging (Fig. 2B, D). These formulations were enveloped and protected by an external pH-sensitive omPEG layer, which can alleviate protein adsorption on the nanoparticle surface and preserve their structural integrity during systemic circulation (Juang et al. 2019). Furthermore, the SLN displayed a positive charge (Fig. S1C), whereas the Lip showed a negative charge (Fig. S1F). Because of the positively charged peptide, both the SLN-HAR and Lip-HAR formulations exhibited an elevated positive charge (Fig. S1B, E, Table 1). The omPEG coating effectively masked the surface charge, contributing to prolonged circulation of the nanoformulation (right y-axis; Fig. 2A, C Table 1). Moreover, the EE% of both HuR CRISPR-loaded SLN and DTX-loaded Lip formulations exceeded 90% (right y-axis; Fig. 2E), with DL% reaching approximately 20% (left y-axis; Fig. 2E).

**Table 1:** Characterization of various SLNs and Lips formulations

Formulations	Average size (nm)	Polydispersity index (PDI)	Zeta potential (mV)
HuR CRISPR/SLN	131.70 ± 1.60	0.21 ± 0.04	18.70 ± 1.40
HuR CRISPR/SLN-HAR	143.10 ± 0.95	0.10 ± 0.02	25.30 ± 0.67
HuR CRISPR/omSLN-HAR	186.30 ± 3.68	0.17 ± 0.02	20.68 ± 1.41
DTX/Lip	144.20 ± 2.93	0.16 ± 0.01	− 16.40 ± 0.15
DTX/Lip-HAR	161.40 ± 8.02	0.15 ± 0.02	− 8.65 ± 0.36
DTX/omLip-HAR	174.10 ± 1.10	0.14 ± 0.01	− 4.26 ± 0.60

### pH-sensitive release, uptake, and intracellular localization of HuR CRISPR/omSLN-HAR and DTX/omLip-HAR

**Fig. 3:**
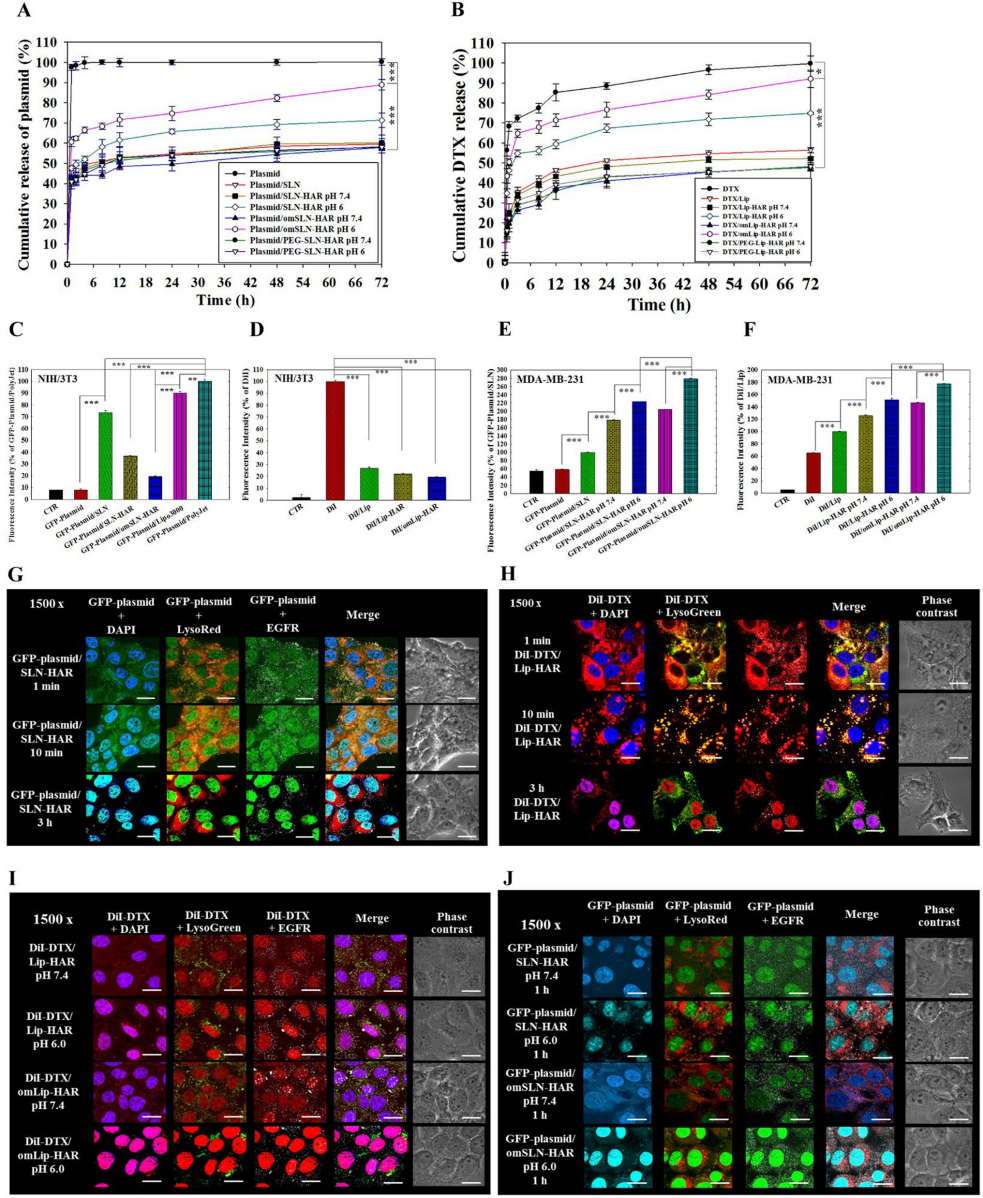
Drug release, cellular uptake, and intracellular localization via pH-sensitive and targeting peptide-modified formulations. **A** In vitro release of HuR CRISPR with or without omSLN-HAR or **B** DTX with or without omLip-HAR at pH 7.4 and 6.0. PEG-SLN-HAR and PEG-Lip-HAR were prepared by substituting DSPE-omPEG into DSPE-PEG (no pH-sensitive bond). **C**–**F** Cellular uptake of GFP-plasmid (GFP-HuR CRISPR plasmid) and DiI-DTX in different formulations in (CD) NIH/3T3 cells at pH 7.4 and (EF) MDA-MB-231 cells at **E** pH 7.4 and (F) 6.0 for 24 h, as analyzed by flow cytometry. Statistical significance at *p < 0.05; **p < 0.01; ***p < 0.001. **G** Intracellular trafficking of GFP-plasmid/SLN-HAR in MDA-MB-231 cells was observed at 1 min, 10 min, and 3 h using CLSM. Blue: DAPI (a nuclear dye); green: GFP-plasmid; red: LysoRed (a lysosome dye); Gray: EGFR (epidermal growth factor receptor). **H** Intracellular trafficking of various DiI-DTX formulations in MDA-MB-231 cells at 1 min, 10 min, and 3 h through CLSM. **I** Cellular internalization of DiI/Lip-HAR and DiI/omLip-HAR in pH 7.4 and 6.0 at 1 h. Blue: DAPI (a nuclear dye); green: LysoGreen (a lysosome dye); red: DiI; gray: EGFR (epidermal growth factor receptor). Magnification: 1500x. **J** Cellular internalization of GFP-plasmid/SLN-HAR and GFP-plasmid/omSLN-HAR at pH 7.4 and 6.0 at 1 h. Blue: DAPI (a nuclear dye); green: LysoRed (a lysosome dye); green: GFP-plasmid; gray: EGFR. Magnification: 1500x

The pH-responsive behavior of omSLN-HAR and omLip-HAR was evaluated by monitoring changes in cumulative release and cellular uptake of HuR CRISPR and DTX at pH 7.4 and 6.0. Figures 3A and B illustrate the release kinetics of HuR CRISPR and DTX from the SLN and Lip formulations at 37 °C under pH 7.4 and pH 6.0 conditions. In the control group, both HuR CRISPR and DTX exhibited faster release than the SLN and Lip formulations. The omSLN-HAR and omLip-HAR formulations elicited sustained release patterns for HuR CRISPR and DTX at pH 7.4. Nevertheless, a notable acceleration in the release of HuR CRISPR and DTX was observed in omSLN-HAR and omLip-HAR at pH 6.0. Under acidic circumstances, HuR CRISPR/omSLN-HAR and DTX/omLip-HAR achieved over 90% release within 72 h. Remarkably, at pH 6.0, there was no discernible difference between free DTX and DTX/omLip-HAR, suggesting nearly complete release from omLip-HAR. In contrast, substituting omPEG with DSPE-PEG5000 to prepare PEG-SLN-HAR or PEG-Lip-HAR, which lacked pH-sensitive imine bonds, did not exhibit a noticeable increase in the release rate at pH 6.0 (Fig. 3A, B).

To verify whether these pH-responsive formulations also enhanced cellular uptake, we substituted HuR CRISPR and DTX with GFP-plasmid and DiI as fluorescent probes. The commercial transfection reagents Lipofectamine™ 3000 and PolyJet™ served as comparisons to the SLN formulations. The minimal uptake of GFP-plasmid/omSLN-HAR and DiI/omLip-HAR in normal NIH/3T3 cells suggests restricted internalization in normal cells (Fig. 3C, D). GFP-plasmid/omSLN-HAR and DiI/omLip-HAR demonstrated the highest cellular uptake in TNBC MDA-MB-231 cells at pH 6.0 (Fig. 3E, F), consistent with the in vitro release results. The intracellular localization of SLN and Lip formulations in MDA-MB-231 cells was also visualized by CLSM. In Fig. 3G, the confocal image displays a faint green signal from the GFP-plasmid co-localizing with EGFR at 1 min. Subsequently, at 10 min, the GFP-plasmid was clearly superimposed with lysosomes, as evidenced by the overlapping green and red fluorescence signals. By the 3-h interval, the GFP plasmid was nearly completely disassociated from the lysosomes and was prominently concentrated within the nucleus, emitting a robust green fluorescence signal (Fig. 3G). In Fig. 3H, we observed scattered DiI-DTX exhibiting a weak red signal at 1 min, followed by the accumulation of DiI-DTX within lysosomes at 10 min. Within 3 h, DiI-DTX almost entirely escaped from the lysosomes. With the assistance of the A peptide, both SLN-HAR and Lip-HAR were effectively targeted and co-localized with EGFR within 1 min, as indicated by clear fluorescence signals (Fig. 3G, H). The R peptide also facilitated the localization of the GFP-plasmid in the nucleus (Fig. 3G), revealing effective nuclear delivery by SLN-HAR.

In Fig. 3I, DiI-DTX was incorporated into Lip-HAR and omLip-HAR to evaluate pH-sensitivity at both pH 7.4 and 6.0 after 1 h. Initially, DiI-DTX in Lip-HAR showed nearly identical fluorescence distribution at both pH levels. However, a notable divergence became clear at pH 6.0, where DiI-DTX/omLip-HAR demonstrated superior lysosomal escape capabilities and clearer red fluorescence signals. These findings align with the earlier uptake results presented in Fig. 3F. We also investigated the intracellular trafficking of unmodified and single-peptide-modified SLN and Lip, as shown in Fig. S2, S3. Unmodified Lip displayed no distinct effects (Fig. S2), whereas DiI-DTX/Lip-H showed higher DiI-DTX accumulation after 1 h at pH 6.0 than that of DiI-DTX/Lip (Fig. S3A). DiI-DTX/Lip-A exhibited a pronounced EGFR-targeting effect (Fig. S3B), and DiI-DTX/Lip-R displayed localization in both the nucleus and cytoplasm at 3 h (Fig. S3C). These results highlight the distinct capabilities of H, A, and R peptides, each of which effectively fulfills their respective functions (Fig. S3). Similarly, the inclusion of the GFP-plasmid into SLN-HAR and omSLN-HAR revealed discernible pH-sensitivity differences following 1-h uptake at pH 7.4 and 6.0 (Fig. 3J).

### Transfection and knockout efficiency of HuR CRISPR in the SLN formulations

**Fig. 4:**
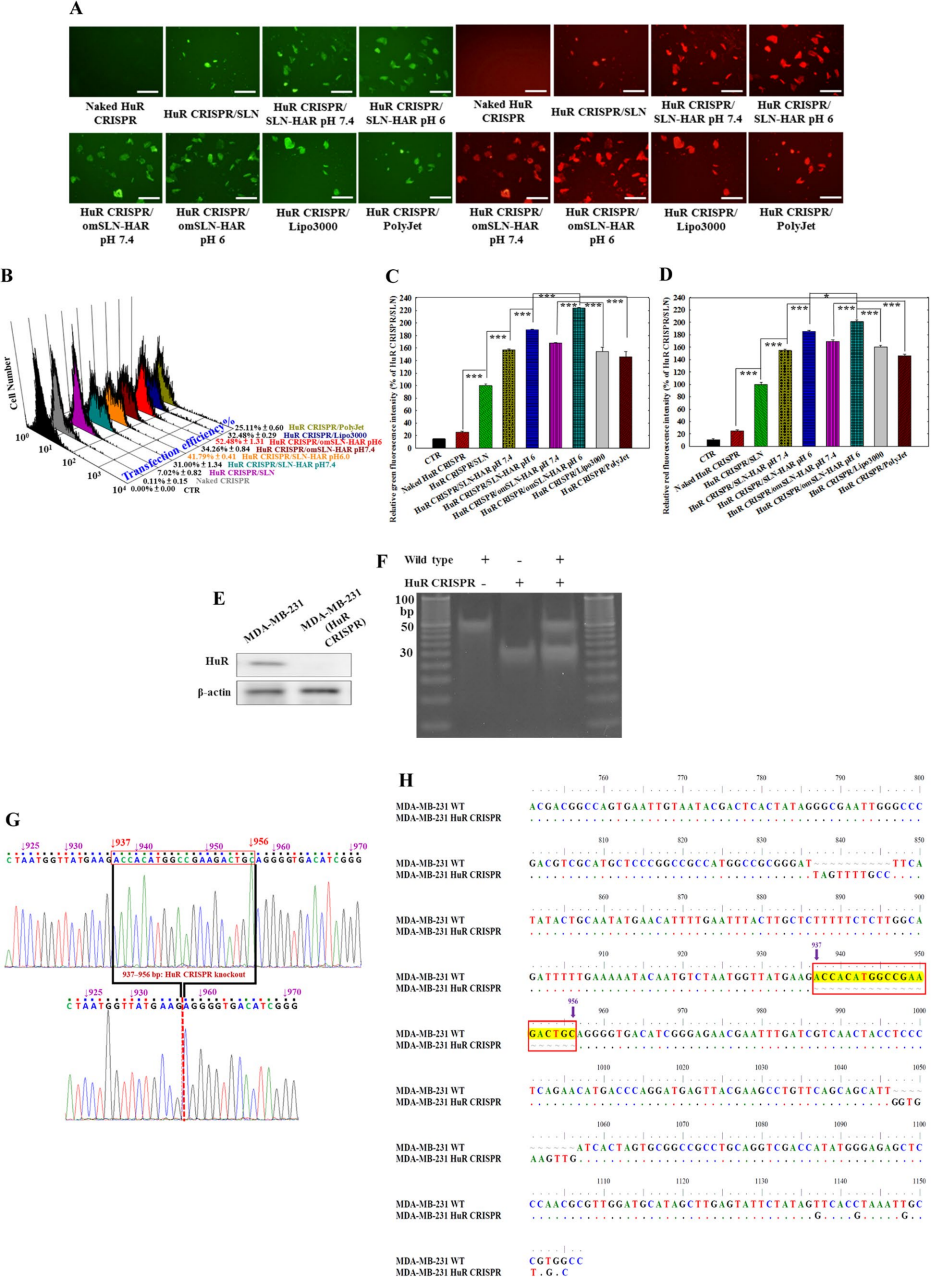
Transfection and HuR knockout efficiency in MDA-MB-231 cells. **A** Fluorescence microscopy showed green fluorescence of transfected cells and HuR knockout red fluorescence in MDA-MB-231 cells. Magnification: 200X. **B** Transfection efficiency of HuR CRISPR in SLN formulations and other commercial agents was measured in MDA-MB-231 cells via flow cytometry. **C**-**D** Fluorescence intensity of (C) green (transfected cells) and **D** red (HuR knocked out cells) in MDA-MB-231 cells quantified by flow cytometry. Statistical significance at *p < 0.05; **p < 0.01; ***p < 0.001. (**E**) HuR was knocked out by the CRISPR/Cas9 system and confirmed by Western blot assay. **F** Double-stranded DNA partial sequences of HuR in MDA-MB-231 and HuR CRISPR MDA-MB-231 cells were verified by PAGE. **G** Verification of nucleic acid sequences was conducted in both wild-type MDA-MB-231 cells (upper panel) and MDA-MB-231 cells with HuR knockout by CRISPR/Cas9 (lower panel, HuR CRISPR). **H** Comparison of gene sequences between MDA-MB-231 wild type (WT) and HuR CRISPR reveals a precise deletion of 937–956 bp in the HuR sequence, highlighted in yellow

Figures 4A–D depict the efficiency of HuR CRISPR transfection and knockout, as assessed using fluorescence microscopy and flow cytometry. A surrogate reporter system was employed to validate the identification of transfected and knockout cells for HuR. Transfected cells exhibited green fluorescence due to the expression of the surrogate plasmid’s GFP, while red fluorescence was absent as the tdTOMATO sequence was out of frame and unable to translate (Fig. 4A, B). If the CRISPR/Cas system effectively knocked out the HuR sequence, the tdTOMATO sequence became in frame, resulting in the emission of red fluorescence, indicating the successful knockout of the HuR sequence in MDA-MB-231 cells (the right panels of Fig. 4A). In Fig. 4A, it is evident that omSLN-HAR at pH 6.0 displayed noticeable emission of both green and red fluorescence. At pH 6.0, omSLN-HAR exhibited the highest transfection and knockout effect in MDA-MB-231 cells, as quantified by flow cytometry, particularly highlighted in Fig. 4B–D. The green fluorescence shows the successful transfection of the surrogate plasmid’s GFP into MDA-MB-231 cells (as depicted in the left panels of Fig. 4A). At pH 6.0, SLN-omHAR exhibited the highest transfection efficiency (percentage normalized per 100 cells), reaching 52.48% ± 1.31 (Fig. 4B; right panels), surpassing SLN-omHAR at pH 7.4, which achieved 34.26% ± 0.84 (Fig. 4B; right panels). In terms of relative transfection comparison of different formulations, SLN-omHAR at pH 6.0 demonstrated the highest percentage of relative green fluorescence intensity among all groups (Fig. 4C), measuring 224.28% ± 0.70, compared to SLN-omHAR at pH 7.4, which achieved 168.14% ± 0.61, and commercial transfection reagents in MDA-MB-231 cells (Fig. 4C). Regarding relative HuR knockout comparison of various formulations, SLN-omHAR also demonstrated an enhanced HuR knockout effect compared to other formulations at pH 6.0, as depicted by the red tdTOMATO fluorescence in the right panels of Fig. 4A. The highest percentage of relative red fluorescence intensity by SLN-omHAR at pH 6.0 among all groups was 201.40% ± 2.27 (Fig. 4D), in contrast to SLN-omHAR at pH 7.4, which achieved 169.34% ± 2.35, and commercial transfection reagents in MDA-MB-231 cells (Fig. 4D). These results emphasize the significant enhancement in pH-responsive transfection efficiency and HuR knockout effect achieved by modifying omPEG and HAR peptides onto SLN (Fig. 4A–D). Furthermore, CLSM results indicated that the GFP-plasmid released from omSLN-HAR at pH 6.0 exhibited incremental green GFP fluorescence, co-localizing with nuclear blue fluorescence (stained by DAPI). This suggests successful endosomal escape of the GFP-plasmid from omSLN-HAR under acidic pH conditions, facilitating its delivery to the nucleus (Fig. 3J).

To confirm successful knockout of the targeted HuR sequence, cellular extracts were prepared from both MDA-MB-231 cells and HuR CRISPR-modified MDA-MB-231 cells for Western blot analysis. As shown in Fig. 4E, the CRISPR/Cas9 system successfully achieved HuR knockout in HuR CRISPR MDA-MB-231 cells. To confirm this, the HuR partial sequence in both MDA-MB-231 and HuR CRISPR MDA-MB-231 cells was selectively amplified using PCR with HuR primers, and the resulting DNA fragments were examined using 12% PAGE. Figures 4F–H and S4 reveal that PAGE and DNA sequencing results indicate a precise 20-base pair difference between MDA-MB-231 cells and HuR CRISPR MDA-MB-231 cells, confirming the targeted deletion by the CRISPR/Cas9 system. This verification establishes the unique characteristics of HuR CRISPR MDA-MB-231 cells for future experiments. Specifically, the sequencing profile of these cells (Figs. 4G, H. S4) demonstrates a homozygous deletion of 937–956 bp in the HuR gene, with no wild-type allele present.

### Cytotoxicity of SLN-HAR and Lip-HAR on normal and TNBC cells

**Fig. 5:**
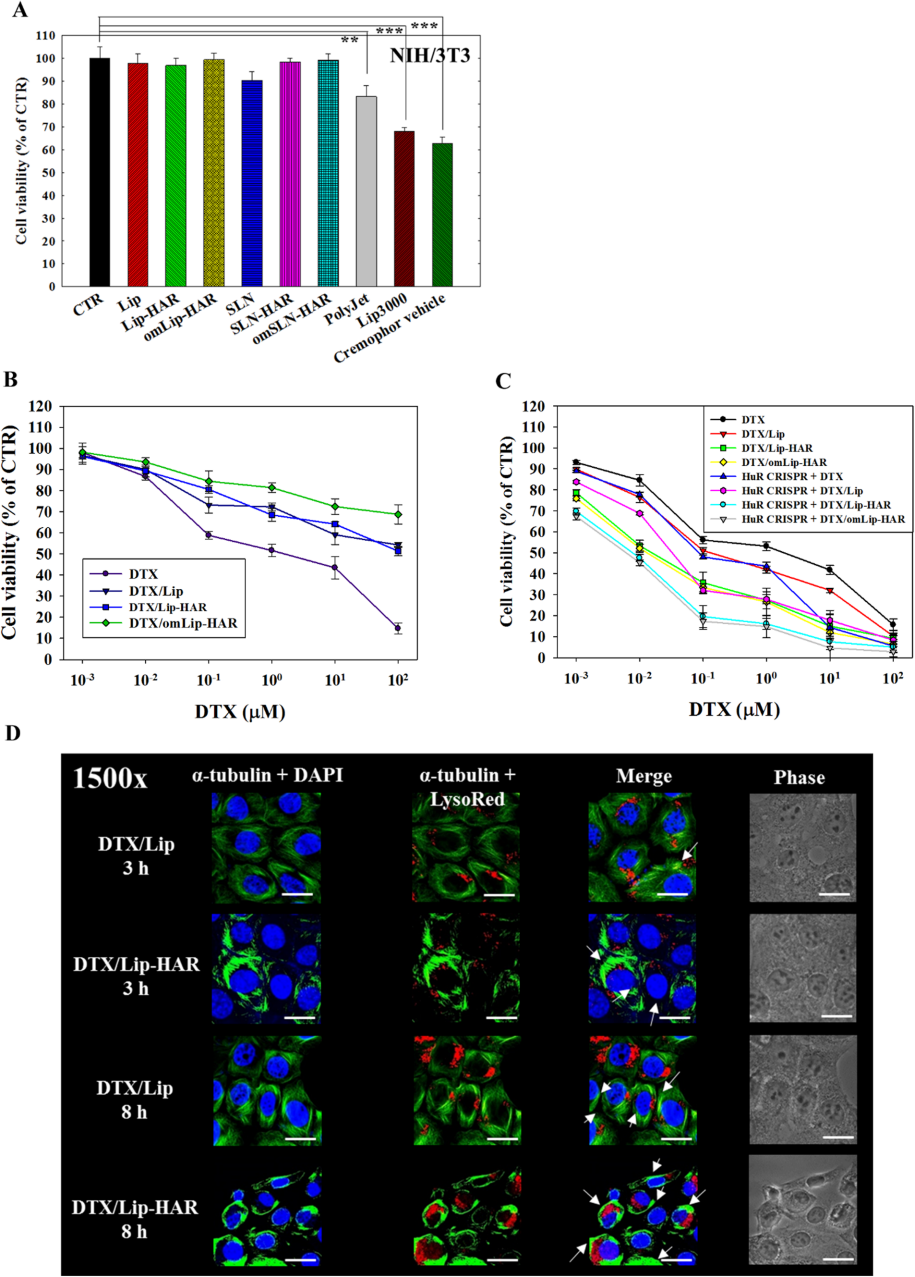
Cytotoxicity and microtubule stabilization of different formulations in MDA-MB-231 and/or NIH/3T3 cells. **A** Evaluation of the cytotoxicity of empty formulations and vehicles on NIH/3T3 cells. (BC) Viability assessment in DTX-loaded formulations using both **B** NIH/3T3 and **C** MDA-MB-231 cells, with or without HuR CRISPR knockout. Cell viability was measured by the sulforhodamine B (SRB) assay. **A**–**C** Statistical significance at *p < 0.05; **p < 0.01; ***p < 0.001. (D) MDA-MB-231 cells were treated with various DTX formulations for 3 h and 8 h to observe the localization and polymerization of microtubules using CLSM. The DTX concentration in all treatments was 0.03 µM (IC30). Magnification: 1500 x

To optimize the anticancer efficacy and reduce potential side effects, we encapsulated DTX within nanoparticles modified with HAR peptide and omPEG. This design enhanced targeting precision and cytotoxicity toward cancer cells while minimizing the impact on normal tissues. The cytotoxicity assessment in Fig. 5A compares the safety of various formulations in noncancerous NIH/3T3 cells. The results revealed that commercial agents and Cremophor®-containing vehicles elicited a cytotoxic effect ranging from 20 to 40% in NIH/3T3 cells, whereas SLN and Lip formulations induced minimal cell death (Fig. 5A). Figures 5B and C depict the evaluation of cell viability in DTX-loaded formulations using both NIH/3T3 and MDA-MB-231 cells with or without HuR CRISPR knockout. Among all groups, DTX/omLip-HAR showed the lowest cytotoxicity in NIH/3T3 cells, possibly due to the shielding provided by omPEG under pH 7.4 conditions (Fig. 5B). As shown in Fig. 5C, DTX-encapsulated liposomes demonstrated higher cytotoxicity than free DTX, particularly in the presence of peptide modifications. DTX/Lip-HAR yielded cell viability results comparable to those of DTX/omLip-HAR, indicating the potential detachment of the omPEG layer in DTX/omLip-HAR, leading to similar in vitro effects. It is worth noting that HuR CRISPR-modified MDA-MB-231 cells exhibited reduced cell viability in cytotoxicity assays under various treatments, as displayed in Figs. 5C and S5. Furthermore, alterations in microtubule morphology were visualized using confocal imaging. As shown in Fig. 5D, the impact of DTX/Lip-HAR was notably superior to that of DTX/Lip. MDA-MB-231 cells treated with DTX/Lip-HAR displayed significantly thicker and more polymerized microtubule fibers at both 3 and 8 h. These results also emphasize the role of the cell-penetrating R peptide in aiding DTX localization within the cytoplasm and stabilizing microtubule fibers (Fig. S6).

### Impact of HuR CRISPR and DTX-loaded formulations on cell cycle and apoptosis in MDA-MB-231 cells

**Fig. 6:**
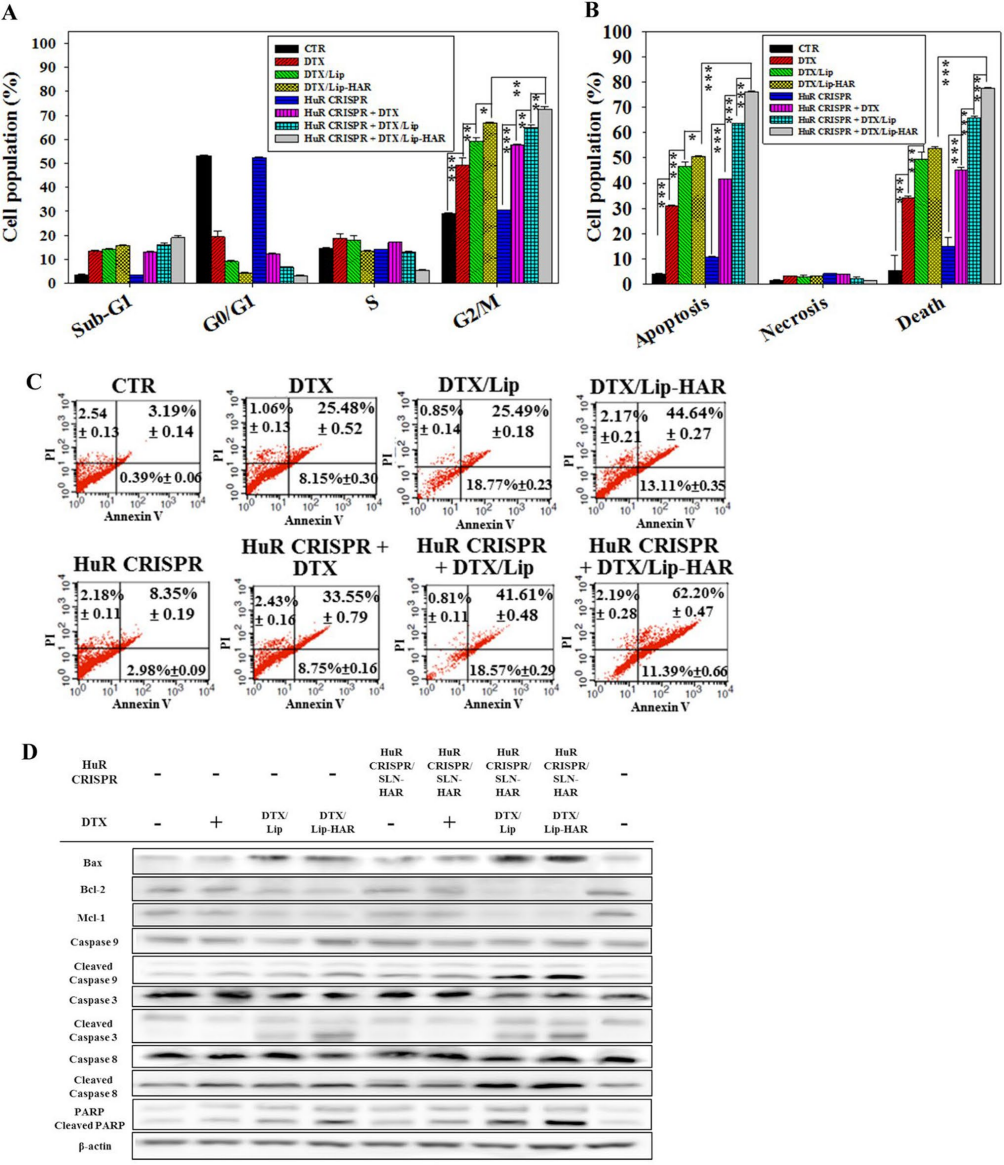
Modulation of the cell cycle and apoptosis in response to various HuR CRISPR and DTX formulations. **A** Cell cycle analysis was performed. **B** Relative percentages, and **C** cell population distribution of apoptosis, necrosis, and cell death in the MDA-MB-231 cell population following a 48-h treatment with DTX (0.03 μM, IC30) in different formulations with or without HuR CRISPR/SLN-HAR were evaluated using Annexin V-FITC/propidium iodide (PI) assay. Distinct cell populations, including viable cells (Annexin V^−^/PI^−^), early apoptotic cells (Annexin V^+^/PI^−^), late apoptotic or necroptotic cells (Annexin V^+^/PI^+^), and dead cells (Annexin V^−^/PI^+^), were identified using a FACSCalibur flow cytometer. **D** Protein expression in the apoptosis-associated pathway was assessed using Western blot analysis

As illustrated in Fig. 6A, DTX/Lip-HAR induced a pronounced G2/M phase arrest (66.79 ± 0.47%), surpassing the DTX group (49.22 ± 3.25%). Remarkably, when administered to HuR-silenced MDA-MB-231 cells, DTX/Lip-HAR demonstrated the most remarkable G2/M arrest among all groups (72.44 ± 1.29%). Moreover, the presence of cells in the sub-G1 phase represents the apoptotic cell population (Fahmy et al. 2023). For DTX/Lip-HAR treatment, the sub-G1 phase population was 15.84 ± 0.10% (in comparison to the control groups at 3.63 ± 0.32%), whereas the combination of HuR CRISPR with DTX/Lip-HAR increased the population to 19.00 ± 1.01%. These findings indicate that HuR deletion effectively enhances apoptosis induction in MDA-MB-231 cells (Fig. 6A).

To further investigate the apoptotic effects of the HuR CRISPR and DTX combination, we employed the annexin V/PI staining assay to determine various cell populations. As illustrated in Fig. 6B and C, treatment with DTX/Lip-HAR in combination with HuR CRISPR/SLN-HAR resulted in a more pronounced apoptotic and cell death effect on MDA-MB-231 cells, with minimal populations showing signs of necrosis. In Fig. 6D and S7A, protein expression in the apoptotic pathway was analyzed. Notably, the levels of apoptotic-related proteins, including Bax, cleaved caspase  -3,  -8, -9, and PARP, were elevated, whereas the levels of the anti-apoptotic proteins Bcl-2 and Mcl-1 declined (Fig. S7A). The DTX formulations not only reduced the viability of MDA-MB-231 cells but also enhanced cytotoxicity when combined with HuR CRISPR/SLN-HAR by inducing apoptotic-associated pathways (Figs. 5C, 6, S7A).

### Diverse pathways regulated by various DTX and HuR CRISPR formulations in MDA-MB-231 cells

**Fig. 7:**
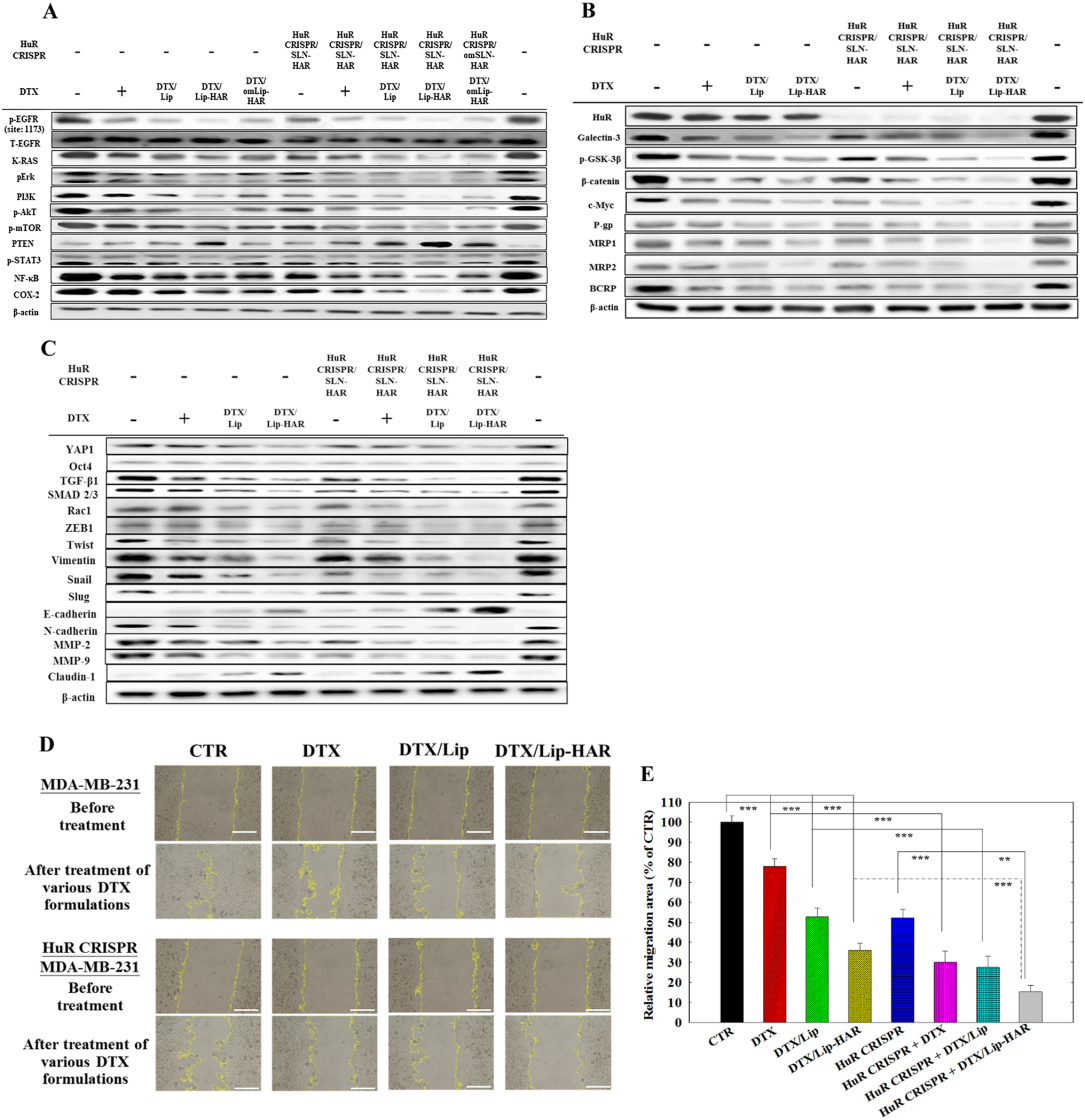
The impact of HuR CRISPR and/or DTX-loaded formulations on multiple signaling pathways was evaluated through the analysis of protein expression, along with the assessment of relative cell migration percentages in MDA-MB-231 cells. Cells with or without HuR CRISPR knockout were treated with different formulations of DTX (2 µM, IC50) for 48 h. Protein expression of **A** EGFR/PI3K/AKT, **B** HuR/galectin-3/GSK-3β/β-catenin, and P-gp/MRPs/BCRP, along with **C** YAP1/TGF-β/ZEB1/Slug/MMP pathways by Western blot assay. **D** Wound-healing assay and **E** migration percentage analysis. For the wound-healing assay, cells were treated with various formulations for 18 h, and migration areas were quantified using ImageJ. Magnification: 200 × (Statistical significance at *p < 0.05; **p < 0.01; ***p < 0.001)

Given that HuR may regulate EGFR pathway signaling through mRNA stabilization and that the A peptide can target EGFR on cells, we verified protein expression by Western blotting, as shown in Fig. 7A. Furthermore, several studies have indicated that docetaxel treatment may induce a decrease in p-EGFR expression in different cancer types, potentially bolstering its anti-tumor properties (Hashemi et al. 2023). Our investigation substantiates this observation, as evidenced by the noteworthy suppression of p-EGFR expression by DTX-loaded formulations (Fig. 7A). In MDA-MB-231 cells, the protein expression of total EGFR (T-EGFR) remained unchanged, while phosphorylated EGFR (p-EGFR) showed a significant reduction after treatment with HuR CRISPR/SLN-HAR and/or DTX-loaded formulations (Fig. S7B). As a consequence of HuR knockout and the resulting reduction in COX-2 expression, multiple EGFR signaling pathways, such as PI3K/p-AkT/mTOR, K-RAS/p-Erk, and p-STAT3/NF-κB, were suppressed. These inhibitory effects were further amplified with the addition of DTX to various formulations, notably through Lip-HAR (Fig. 7A, S7B).

In Fig. S8A, the mRNA associated with galectin-3/β-catenin/c-Myc were all declined after HuR knockout in MDA-MB-231 cells. The mRNA levels of pivotal constituents in the MDR pathway, encompassing P-gp, MRP1, and MRP2, were diminished as a result of the suppression in the HuR-associated β-catenin pathway, as illustrated in Fig. S8B. The Western blot assay also illustrated the correlation between the HuR/galectin-3/GSK-3β/β-catenin and P-gp/MRPs/BCRP pathways (Figs. 7B, S7C, D). The combined treatment involving HuR CRISPR and DTX formulations enhanced the effectiveness of reducing mRNA and protein levels in the GSK-3β/β-catenin and MDR pathways (Figs. 7B, S7C, D, S8A, B). The combined therapy, especially HuR CRISPR/SLN-HAR and DTX/Lip-HAR, may confer reduced drug resistance.

In Fig. 7C and S7E, the western blot assay demonstrated that DTX formulations, along with HuR CRISPR, led to a decrease in the protein expression of the EMT-associated pathway. Furthermore, HuR CRISPR-modified MDA-MB-231 cells demonstrated additional reductions in the protein expression levels of TGF-β, SMAD 2/3, ZEB1, Snail, YAP1, and Oct4 upon co-administration of DTX formulations (Fig. 7C, S7E, F). Moreover, various EMT-associated proteins, such as Twist, vimentin, Slug, E-cadherin, N-cadherin, MMP-2, MMP-9, and claudin-1, exhibited notable inhibition to varying degrees following treatment with HuR CRISPR and/or DTX in different formulations (Fig. 7C, S7E, F). Cell migration, a pivotal component of cancer cell behavior within the EMT pathway, was evaluated by quantifying the wound healing capability of MDA-MB-231 cells, both with and without HuR CRISPR, following an 18-h DTX treatment (Fig. 7D). As displayed in Fig. 7E, the co-administration of HuR CRISPR/SLN-HAR and DTX/Lip-HAR resulted in the most significant suppression of migratory capabilities (84.71 ± 3.15%) and mitigated the invasive characteristics of MDA-MB-231 cells compared with DTX/Lip-HAR alone (64.00 ± 3.63%).

### In vivo PET/CT imaging, antitumor efficacy, and biodistribution studies

**Fig. 8:**
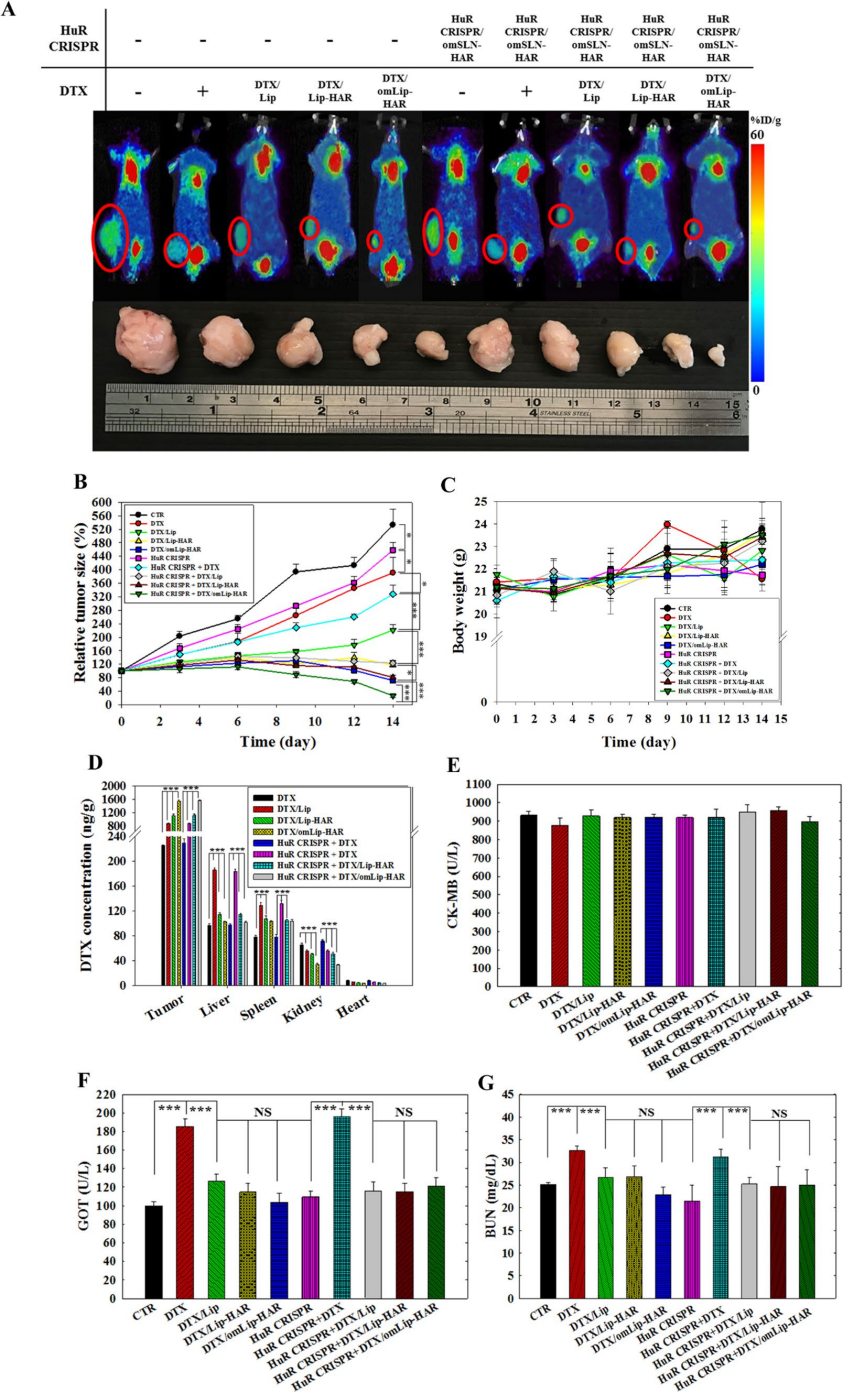
Antitumor efficacy and safety of different DTX formulations with or without HuR CRISPR knockout in MDA-MB-231-bearing mice. Mice carrying MDA-MB-231 cells, either with or without the HuR deletion, were injected intravenously with various DTX formulations. **A** PET/CT scans taken at the end of the fourteenth day of therapy of representative mice from each group. Tumor: red circles. The radioactivity levels within the tissue and tumor were quantified as the uptake values per administered dose of the probe, denoted as a percentage of injected dose per gram of tissue (%ID/g). **B** Digital calipers were used to assess tumor size. The relative tumor size (%) is calculated by dividing the tumor volume at a specific time point by the initial tumor volume (Day 0) for each experimental group, and then multiplying the quotient by 100% to express the change relative to the baseline (Day 0). Statistical significance at *p < 0.05; **p < 0.01; ***p < 0.001. **C** Body weight was monitored every 3 days and reported as a weight scale (g). **D** A biodistribution analysis of various DTX formulations was performed in MDA-MB-231- and HuR CRISPR MDA-MB-231-bearing mice, and the concentration of DTX was determined by HPLC. (E–G) FUJI DRI-CHEM 4000i was used to measure the biochemical indicators of the **E** heart, **F** liver, and **G** kidney of mice treated with various formulations (Statistical significance at *p < 0.05; **p < 0.01; ***p < 0.001; NS: not significant *p* > 0.05)

MDA-MB-231 tumor-bearing mice with or without HuR CRISPR knockout were constructed to assess the in vivo antitumor efficacy of various DTX formulations (5 mg/kg body weight of DTX). As depicted in Fig. 8A, tumor-bearing mice treated with different DTX formulations exhibited varying degrees of reduction in ^18^F-FDG signals around the tumor. Among these, HuR CRISPR-modified MDA-MB-231 tumor-bearing mice treated with DTX/omLip-HAR demonstrated the most significant reduction in ^18^F-FDG retention, resulting in a tumor size reduction to 26.57 ± 4.90% of the original volume at the end of the 14-day treatment (Fig. 8B). To ensure safety, we evaluated body weight changes and observed that the administration of DTX-loaded formulations with or without HuR CRISPR knockout to MDA-MB-231-bearing mice did not result in significant alterations in body weight (Fig. 8C). Furthermore, the biodistribution of DTX in various formulations in MDA-MB-231-bearing mice was measured (Fig. 8D). Liposomal DTX formulations were predominantly detected in the tumor tissues of both MDA-MB-231 and HuR CRISPR MDA-MB-231-bearing mice, with DTX/omLip-HAR exhibiting the highest accumulation among all the groups.

### Evaluation of in vivo biosafety using biochemical tests, blood cell counts, and H&E staining in MDA-MB-231-bearing mice

**Fig. 9:**
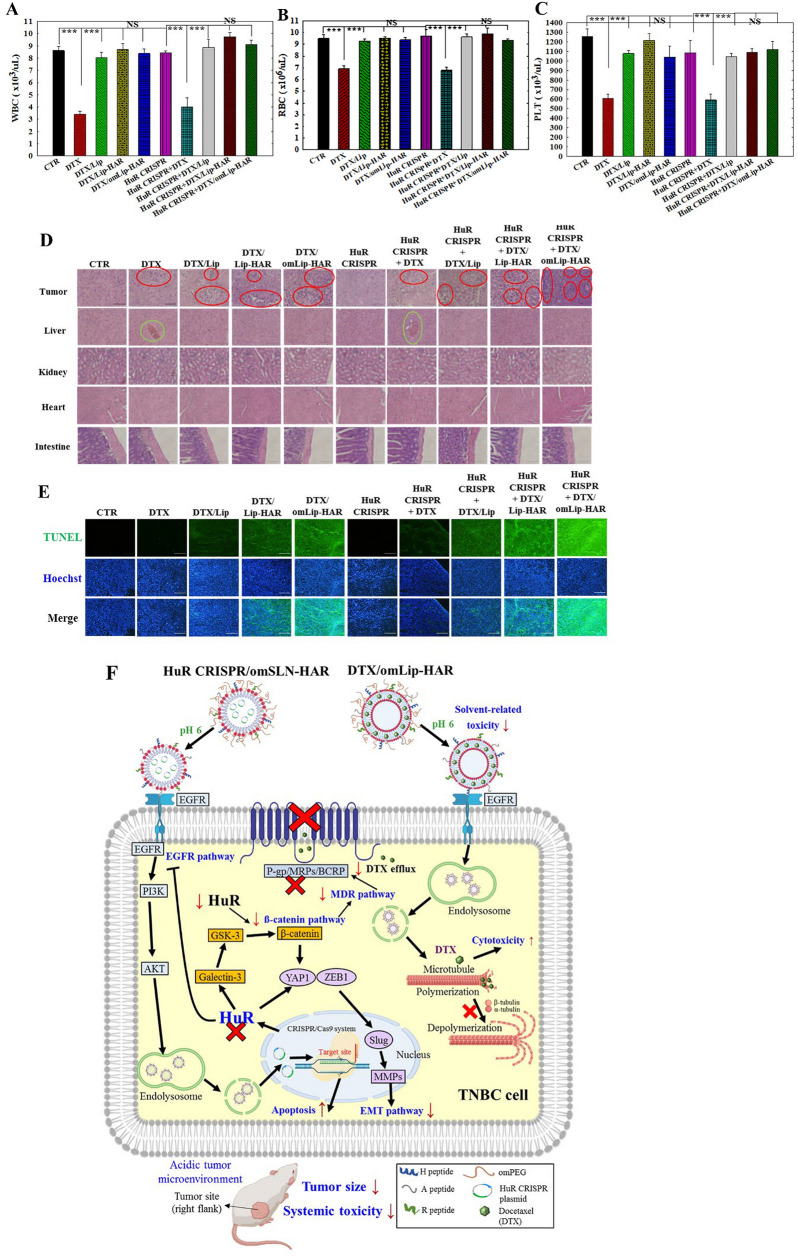
Comprehensive scheme and assessment of in vivo biosafety: blood cell counts and H&E staining analysis in MDA-MB-231-bearing mice. Blood indexes such as **A** white blood cells, **B** red blood cells, and **C** platelets were measured by Sysmex XT-1800iv (***p < 0.001 compared with CTR group. ^†^p < 0.05; ^††^p < 0.01; ^†††^p < 0.001 compared with DTX group. ^§^p < 0.05; ^§§^p < 0.01; ^§§§^p < 0.001 compared with HuR CRISPR + DTX group). **D** Photomicrographs depicting H&E staining of mouse tissues from tumor, liver, kidney, heart, and intestinal sections. Red circles indicate necrosis or apoptosis. Green circles denote cell injury. **E** TUNEL analysis in MDA-MB-231-tumor-bearing mice (green) on the last day of treatment. Nuclei (blue) were stained with Hoechst. **F** The scheme illustrates how co-treatment with HuR CRISPR/omSLN-HAR and DTX/omLip-HAR modulates multiple pathways in an acidic tumor microenvironment through pH-sensitive omPEG decoating. The CRISPR/Cas9 editing system targeted the HuR gene, resulting in the suppression of EGFR, Wnt/β-catenin, MDR, and EMT pathways. This co-treatment influenced key signaling pathways, including EGFR/PI3K/AKT, HuR/galectin-3/GSK-3β/β-catenin, P-gp/MRPs/BCRP, and YAP1/TGF-β/ZEB1/Slug/MMPs, in TNBC-bearing mice, facilitating DTX accumulation, promoting cancer cell death, suppressing tumor growth, and reducing systemic toxicity.

Typical biochemical indexes, such as CK-MB for heart function, GOT for liver function, BUN for kidney function (Fig. 8E–G), and blood cell counts for WBC, RBC, and platelets (Fig. 9A–C), were evaluated in blood samples from MDA-MB-231- and HuR CRISPR MDA-MB-231-bearing mice following various treatments. In comparison with the control groups, neither DTX nor other formulations showed significant alterations in the serum levels of CK-MB, as depicted in Fig. 8E, indicating the negligible heart toxicity of all formulations. The serum levels of GOT and BUN significantly increased following the administration of DTX or DTX + HuR CRISPR (p < 0.001) and subsequently returned to their baseline levels after loading into liposomal formulations (Fig. 8F, G). This implies that DTX in Lip, Lip-HAR, or omLip-HAR could reduce the degree of toxicity. Figures 9A–C illustrate the restoration of reduced blood cell counts caused by DTX or DTX + HuR CRISPR-induced bone marrow suppression to normal values following treatment with DTX in Lip or Lip-HAR formulations, both with and without HuR CRISPR. This demonstrates the absence of in vivo hematotoxicity. Furthermore, we performed a histological assessment using H&E staining to observe potential biosafety concerns within the tissues. Among all the treatment groups, the tumor tissues in the HuR CRISPR + DTX/omLip-HAR group exhibited the most pronounced nuclear signs of chromatin condensation (karyopyknosis; highlighted by red circles), indicative of apoptosis and necrosis within the tumors (Fig. 9D, the first panel). Liver injury (emphasized by green circles) was evident in both the DTX and DTX + HuR CRISPR groups (Fig. 9D, the second panel). This tissue damage induced by DTX was correlated with elevated GOT levels, as shown in Fig. 8F. After various treatments, H&E staining of the heart, renal, and intestinal tissues (Fig. 9D, third to fifth panels) did not reveal any significant signs of tissue injury. We also conducted a TUNEL assay to confirm the apoptotic effects within the tumors. As illustrated in Fig. 9E, the combination of HuR CRISPR with DTX/omLip-HAR displayed the highest intensity of green fluorescent signals among all the groups, signifying the most potent induction of tumor-specific apoptosis in MDA-MB-231-bearing mice.

## Discussion

DTX, a cytotoxic taxane, destabilizes microtubules, leading to cell cycle arrest and eventual cancer cell apoptosis (van Eijk et al. 2022; Chaurawal and Raza 2023). Nevertheless, it still presents certain limitations, such as neutropenia and the potential for vomiting (Gupta et al. 2022). Furthermore, the pervasive development of multidrug resistance (MDR) intensifies the obstacle to DTX’s anticancer efficacy against TNBC (Seneme et al. 2022). In addition, the CRISPR/Cas system faces challenges, including rapid degradation, off-target side effects, and limited cellular uptake capacity (Wang et al. 2021). Thus, it is imperative to engineer delivery systems with cancer-targeting capabilities, improved tumor penetration, efficient cellular internalization, escape from endosomes, and effective nuclear localization. These factors are pivotal for achieving superior uptake of both DTX and HuR CRISPR, consequently augmenting the efficacy of TNBC treatment, as depicted in Fig. 1A. The results presented in Figs. 1B–E, 2, and Table 1 illustrate the successful incorporation of these meticulously crafted peptides into lipids, enabling the formulation of nanoparticles with not only high encapsulation and drug loading efficiencies but also a uniform size distribution. Furthermore, our findings highlight the pH-responsive release and uptake capabilities of H peptide and omPEG in an acidic environment compared with neutral pH conditions (Fig. 3, S2, S3). At pH 6.0, the pH-sensitive H peptide altered its conformation (Wang et al. 2021), facilitating penetration into MDA-MB-231 cells and subsequent release of DiI-DTX for cytoplasmic distribution (Fig. S3A). In Figure S3B, the EGFR targeting ability of the A peptide is affirmed, indicating superior targeting of DiI-DTX via Lip-A to EGFR in MDA-MB-231 cells, particularly evident at 10 min post-uptake as detected by CLSM. Our selection of the A peptide was based on its demonstrated ability to target EGFR (Han et al. 2013; Zhou et al. 2019; Williams et al. 2020). Preliminary findings showed varying binding affinities across cancer cell lines for EGFR-binding A, C, and P peptides (Han et al. 2013; Zhou et al. 2019; Williams et al. 2020; Wang et al. 2021; Li et al. 2024): A peptide bound best to TNBC cells (Han et al. 2013; Zhou et al. 2019; Williams et al. 2020); unpublished data), P peptide showed higher affinity in head and neck cancer cells (Wang et al. 2021), and C peptide excelled in pancreatic cancer cells (Li et al. 2024). Variability in peptide targeting efficiency arises from tumor cellular heterogeneity (Zhang et al. 2022; Sadida et al. 2024). Diverse cancer cell subpopulations with distinct profiles differ in receptor expression, signaling, and metabolism, affecting peptide binding and internalization (Yuan et al. 2023; Sadida et al. 2024). Additionally, receptor accessibility within the tumor microenvironment impacts targeting efficiency (Chehelgerdi et al. 2023; Wang et al. 2023a). Understanding these complexities is vital for optimizing peptide-based targeting strategies (Chehelgerdi et al. 2023; Wang et al. 2023a; Yuan et al. 2023; Sadida et al. 2024). Additionally, the intracellular localization of GFP-plasmid/SLN-R into the nucleus is validated in Fig. S3C, most prominently observed at the 3-h time point. Therefore, the combination of omPEG and H peptide along with A and R peptides in omSLN-HAR and omLip-HAR demonstrated superior drug release and uptake profiles in acidic TME (Fig. 3, S2, S3).

Cationic nanoparticles, including SLN formulations, offer an excellent option for delivering anionic gene products such as miR and DNA plasmids (Wang et al. 2021; Li et al. 2024). The results in (Figs. 1, 2 and 3) emphasize the significance of the dual modification, incorporating pH-sensitive omPEG and the H peptide, which enables a response to the acidic TME. Furthermore, the guiding roles of A and R peptides in directing the payload to EGFR and the nucleus serve to enhance cellular uptake, transfection capability, and CRISPR knockout efficiency (Figs. 3, 4). In this study, we employed a comprehensive range of methodologies to assess our findings of a successful HuR knockout. Specifically, we utilized microscopic analysis (Fig. 4A), flow cytometry data (Fig. 4B–D), HuR expression profiling via Western blot (Fig. 4E, F), and sequencing data (Fig. 4G, H, S4). Furthermore, changes in the HuR gene sequence (Figs. 4G, H, S4) also altered the HuR expression profile (Fig. 4E, F). By conducting a thorough comparison of gene sequences from cells analyzed via complete nucleic acid sequencing, we observed a successful deletion of HuR, resulting in an effective HuR knockout, as depicted in Figs. 4G, H, and S4. This evidence in Figs. 4E–G and S4 strongly indicates the precise excision of 937–956 bp in the HuR sequence by the CRISPR/Cas9 system, which solidifies the unique attributes of HuR CRISPR MDA-MB-231 cells and provides a strong foundation for subsequent experiments.

It’s noteworthy that HuR CRISPR-modified MDA-MB-231 cells demonstrated diminished cell viability in cytotoxicity assays across different treatments, as illustrated in Fig. 5C and S5. This result can be attributed to the regulatory influence of HuR on the proliferation of MDA-MB-231 cells (Wang et al. 2023b) for the decrease in cell viability after HuR deletion. Furthermore, DTX stabilizes microtubule polymerization, forming dense microtubule bundles that prevent disassembly, thereby leading to cell cycle arrest and apoptosis (Bhattarai et al. 2022). MDA-MB-231 cells treated with DTX/Lip-HAR exhibited notably robust alterations, displaying prominently thicker and more polymerized microtubule fibers at both 3 and 8 h (Fig. 5D). These compelling findings emphasize the pivotal role of the cell-penetrating R peptide, as vividly illustrated in Fig. S6, in facilitating DTX localization within the cytoplasm and fortifying microtubule stability.

Moreover, the HuR CRISPR and DTX combination enhanced apoptosis in MDA-MB-231 cells, as evidenced by elevated levels of apoptotic-related proteins (Bax, cleaved caspase-3, -8, -9, and PARP) and decreased levels of anti-apoptotic proteins (Bcl-2 and Mcl-1), as depicted in Fig. 6 and S7A. DTX formulations not only reduce cell viability but also augment cytotoxicity when combined with HuR CRISPR (Fig. 5C, 6). Because HuR can regulate EGFR pathway signaling via mRNA stabilization and considering that the A peptide can target EGFR on cells, we confirmed protein expression of EGFR-mediated pathways through Western blotting, as illustrated in Fig. 7A. Furthermore, within the PI3K/Akt/NF-κB signaling pathway, HuR has been identified as a regulator of NF-κB and COX-2 (Zhang et al. 2019a; Shao et al. 2020). This implies that the deletion of HuR in our study can robustly suppress PI3K/Akt/NF-κB/COX-2, a hypothesis confirmed through our investigations (Fig. 7A, S7B). Remarkably, COX-2 plays a role in metabolizing arachidonic acid, converting it into prostaglandin E2 (PGE2), and subsequently activating the EGFR signaling pathway (Lai et al. 2022). Accordingly, the repercussions of HuR knockout led to a substantial decrease in COX-2 expression. Disruption of the EGFR pathway triggered a coordinated downregulation of its downstream signaling cascades, including PI3K/p-Akt/mTOR, K-RAS/p-Erk, and p-STAT3/NF-κB (Fig. 7A, S7B). Notably, the incorporation of DTX into formulations, particularly Lip-HAR, demonstrably potentiated its inhibitory effects (Fig. 7A, S7B).

Previous studies indicated that HuR could target to β-catenin and c-Myc in galectin-3/β-catenin-associated pathway (Lin et al. 2017; Eapen et al. 2019). We validated that the integrated regimen, particularly with HuR CRISPR/SLN-HAR and DTX/Lip-HAR, possesses a compelling capacity to diminish drug resistance by markedly suppressing mRNA and protein levels within pivotal Wnt/β-catenin and MDR pathways, notably galectin-3/GSK-3β/β-catenin and P-gp/MRPs/BCRP (Fig. 7B, S7C, D, S8). Additionally, TGF-β1, SMAD 2/3, ZEB1, and Snail, all integral to the EMT pathway, have been linked to HuR regulation (Eapen et al. 2019; Zhang et al. 2019b; Li et al. 2022). It is noteworthy that Yes-associated protein 1 (YAP1) and octamer-binding transcription factor 4 (Oct4) jointly upregulate EMT-activating factors (Bora-Singhal et al. 2015; Chang et al. 2022), with YAP1 overexpression linked to tumorigenicity, metastasis, and chemoresistance in CRC cells (Li et al. 2018; Ji et al. 2023). Remarkably, HuR can bind and enhance the stability of YAP1, ultimately activating downstream targets, including c-Myc and Slug (Jing et al. 2023). Through rigorous validation using Western blot assays, we substantiated that DTX formulations, in conjunction with HuR CRISPR, induced a compelling reduction in protein expression across critical EMT- and metastasis-associated pathways. The impacted pathways included TGF-β, SMAD 2/3, ZEB1, Snail, YAP1, Oct4, Twist, vimentin, Slug, E-cadherin, N-cadherin, MMP-2, MMP-9, and claudin-1 (Figs. 7C–E, S7E, F).

HuR silencing in MDA-MB-231 cells via HuR CRISPR/SLN-HAR, coupled with DTX/Lip-HAR co-treatment, led to a remarkable breakthrough in antitumor therapeutic efficacy in MDA-MB-231-bearing mice (Fig. 8A, B). This was further corroborated by PET/CT images, which clearly demonstrated that the combined treatment of HuR CRISPR/SLN-HAR and DTX/Lip-HAR outperformed all other treatments, demonstrating superior antitumor efficacy (Fig. 8B). The collaborative effect of DTX/Lip-HAR and HuR CRISPR/SLN-HAR triggered apoptosis in a significantly higher number of tumor cells than any other treatment, as evidenced in Fig. 8E. Importantly, this potent antitumor effect was achieved without causing any significant differences in the body weights of the tumor-bearing mice across all groups (Fig. 8C). The biodistribution results indicated that DTX/Lip without modification primarily accumulated in the liver and spleen, likely due to the reticuloendothelial system. However, DTX in Lip, Lip-HAR, or omLip-HAR were able to circumvent this issue, thereby reducing the degree of toxicity in the liver, kidneys, and spleen (Fig. 8E–G). Figures 9A–C illustrate the restoration of diminished blood cell counts resulting from bone marrow suppression induced by DTX or the combination of DTX and HuR CRISPR. This reversal to normal values was observed after administering DTX in Lip, Lip-HAR, or omLip-HAR formulations, regardless of the presence or absence of HuR CRISPR. The compelling imagery emphasizes the effectiveness of these interventions in rectifying the adverse effects on blood cell counts. All findings in Figs. 8 and 9 highlight the efficacy and safety of these formulations, paving the way for their potential use in future therapeutic applications.

Collectively, Fig. 9F summarize the modulation by HuR CRISPR/omSLN-HAR and DTX/omLip-HAR in an acidic TME via the pH-sensitive de-coating of the omPEG layer. The CRISPR/Cas9 technology was employed to delete the HuR gene, which led to the suppression of EGFR, Wnt/β-catenin, MDR, and EMT pathways. This, in turn, facilitated the accumulation of DTX in the cytoplasm, ultimately resulting in cancer cell death, effective tumor suppression, and reduced systemic toxicity. Furthermore, Fig. 9F depict multiple signaling pathways, including EGFR/PI3K/AKT, HuR/galectin-3/GSK-3β/β-catenin, and P-gp/MRPs/BCRP, as well as YAP1/TGF-β/ZEB1/Slug/MMPs, which were modulated by the co-treatment of HuR CRISPR/omSLN-HAR and DTX/omLip-HAR in TNBC-bearing mice.

In conclusion, under the treatment of DTX/omLip-HAR combined with HuR CRISPR, cell growth in TNBC cells and tumor-bearing mice was significantly reduced, and anticancer efficacy and safety were favorably increased in tumor-bearing mice. With the appropriate modification of pH-sensitive omPEG and functional HAR peptides, the nanoparticles could exert a better effect in targeting and delivering the cargoes to tumor sites. Successful delivery of the HuR CRISPR plasmid via omSLN-HAR and DTX by omLip-HAR could regulate apoptosis, EGFR, Wnt-activated, MDR, and EMT signaling pathways, and consequently effectively inhibit tumor growth and reduce systemic adverse effects in TNBC in vitro and in vivo.

## Supplementary Information

Below is the link to the electronic supplementary material.

Supplementary file1 (1604 KB)

## Data Availability

The data utilized in this study are considered confidential.
