# Supplementary material for: Modulating versatile pathways using a cleavable PEG shell and EGFR-targeted nanoparticles to deliver CRISPR-Cas9 and docetaxel for triple-negative breast cancer inhibition
Source: Arch Pharm Res. 2024 Nov 1;47(10-11):829–53. doi: 10.1007/s12272-024-01514-0 (PMC11602847; doi:10.1007/s12272-024-01514-0)
Supplement: Supplementary file 1 — Supplementary file1 (1604 KB) [file 12272_2024_1514_MOESM1_ESM.pdf]

Supplementary Materials

Modulating versatile pathways using a cleavable PEG shell and EGFR-targeted nanoparticles to deliver CRISPR-Cas9 and docetaxel for triple-negative breast cancer inhibition

Yu-Li Lo[1,2]*, Ci-Jheng Hong[1,3], Chen-Shen Wang[1], Ching-Ping Yang[1]

[1] Institute of Pharmacology, National Yang Ming Chiao Tung University, Taipei 112, Taiwan

[2] Faculty of Pharmacy, National Yang Ming Chiao Tung University, Taipei 112, Taiwan

[3] Department of Pharmacy, Antai Medical Care Corporation Antai Tian-Sheng Memorial Hospital, Pingtung, Taiwan

Running title*: Nanoparticles of HuR CRISPR and Docetaxel for TNBC*

Correspondence: Yu-Li Lo, Institute of Pharmacology, National Yang Ming Chiao Tung University, Taipei 112, Taiwan, Tel +886-228-267-000 (ext. 66451), Email: yulilo@nycu.edu.tw

*Mailing address for Prof. Yu-Li Lo:

Yu-Li Lo, Ph.D.

Distinguished Professor, Institute of Pharmacology/Co-faculty of Pharmacy

National Yang Ming Chiao Tung University

No.155, Sec.2, Linong Street

Taipei 112304, Taiwan

TEL: +886-2-2826-7095

E-mail: yulilo@nycu.edu.tw; lohograce@gmail.com

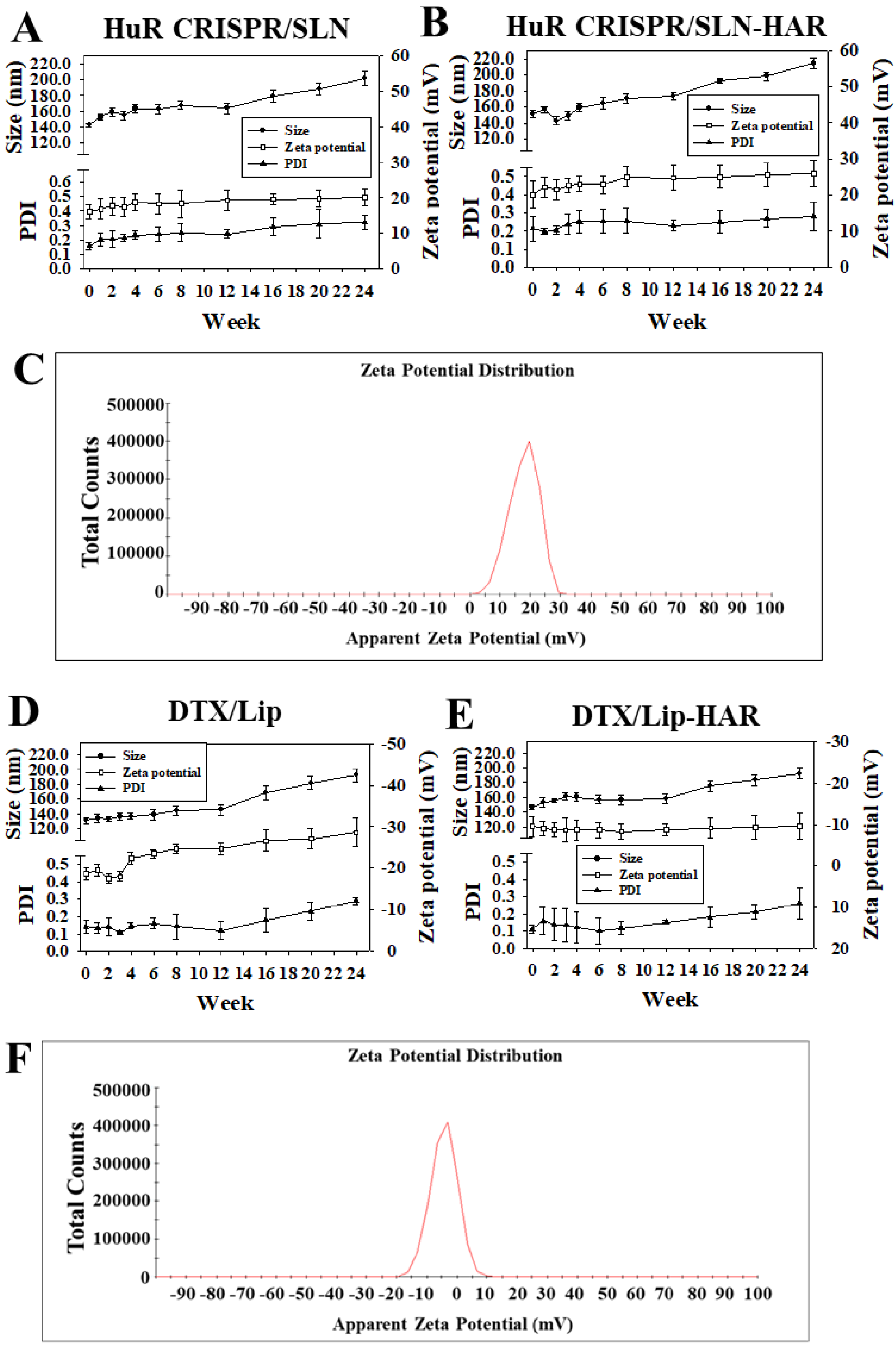

Figure S1. (A-B) Long-term stability of (A) HuR CRISPR/SLN and (B) HuR CRISPR/SLN-HAR; (C) Zeta potential of HuR CRISPR/omSLN-HAR. (D-E) Long-term stability of (D) DTX/Lip and (E) DTX/Lip-HAR. (F) Zeta potential of DTX/omLip-HAR. For each group, n=3.

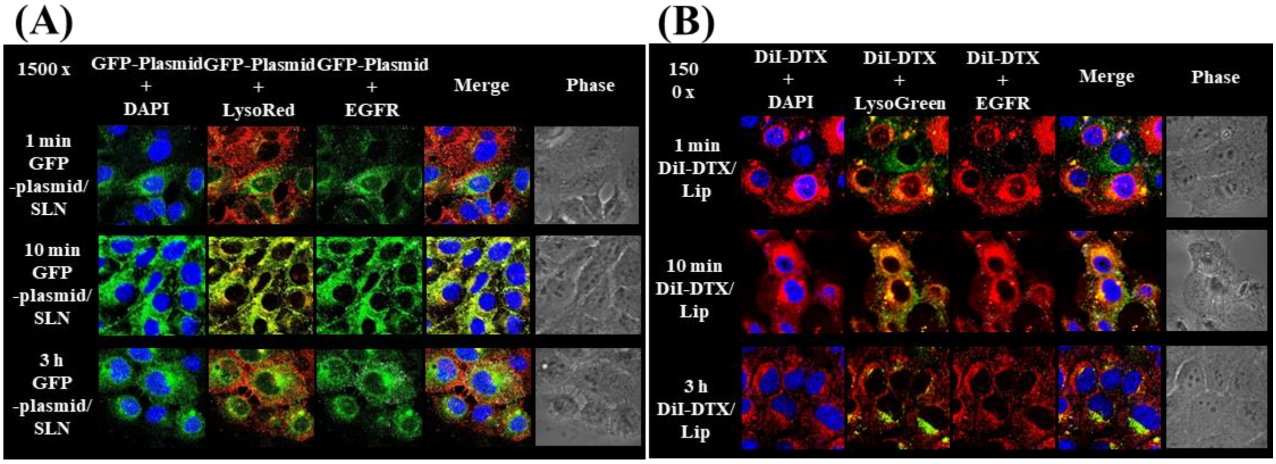

Figure S2. (A) GFP-plasmid/SLN and (B) DiI-DTX/Lip were added to cells for 1 min, 10 min, and 3 h. Intracellular localization of individual formulations in MDA-MB-231 cells detected by CLSM. (A) Blue: DAPI (a nuclear dye); green: GFP-Plasmid; red: LysoRed (a lysosome dye); gray: EGFR (epidermal growth factor receptor). (B) Blue: DAPI (a nuclear dye); green: LysoGreen (a lysosome dye); red: DiI-DTX; gray: EGFR (epidermal growth factor receptor). Magnification: 1500x.

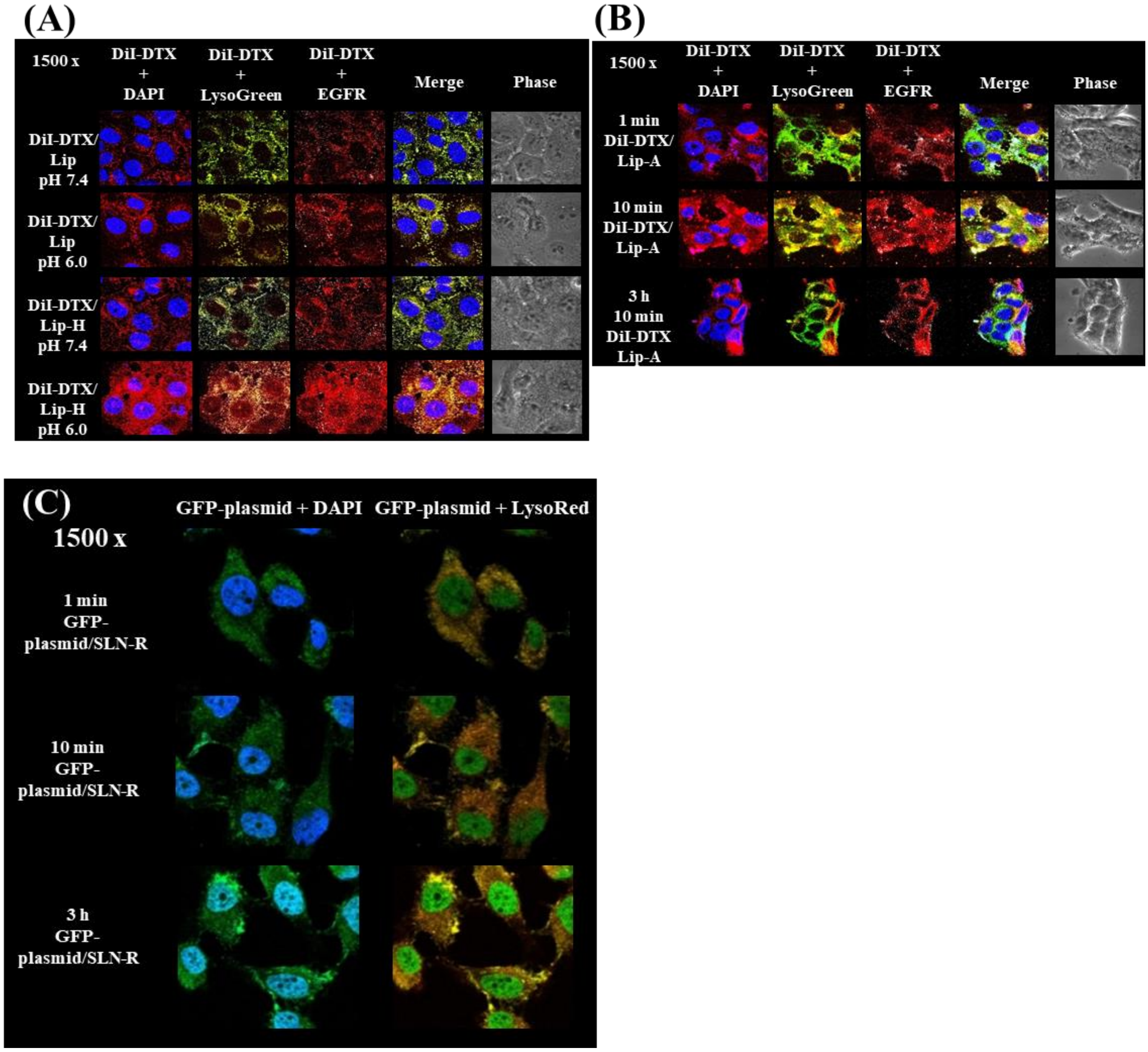

Figure S3. (A) DiI/Lip-H was added to the cells for 1 h at pH 7.4 or 6.0. (B) DiI/Lip-A or (C) GFP-plasmid/SLN-R were added to the cells for 1 min, 10 min, and 3 h. Intracellular localization of the respective DiI-DTX or GFP-plasmid formulations in MDA-MB-231 cells was observed by CLSM. (A) Blue: DAPI (a nuclear dye); green: LysoGreen (a lysosome dye); red: DiI-DTX; gray: EGFR (epidermal growth factor receptor). (B) Blue: DAPI (a nuclear dye); green: GFP-plasmid; red: LysoRed (a lysosome dye). Magnification: 1500x.

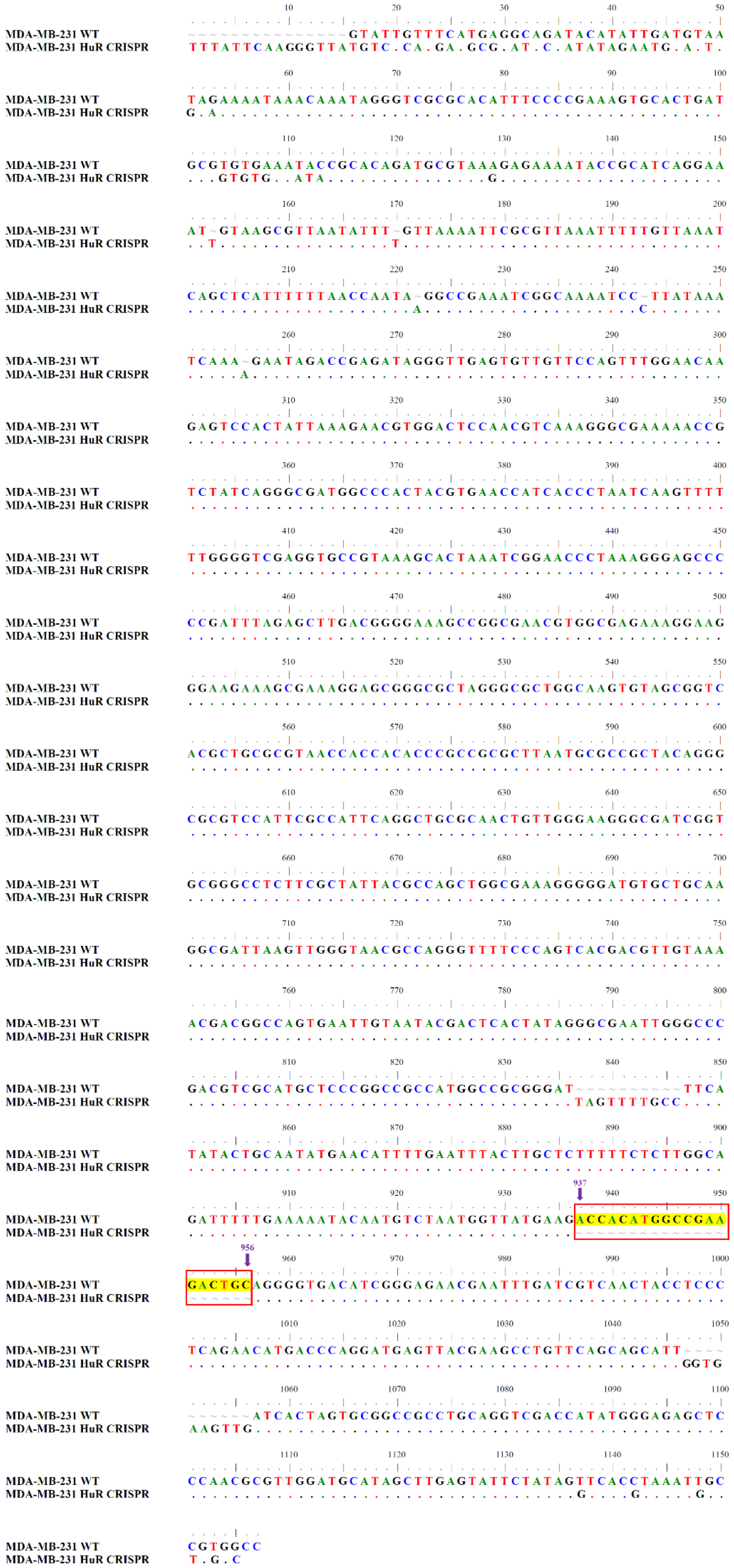
MDA-MB-231 WT
MDA-MB-231 HuR CRISPR
937
956

Figure S4. Comparison of gene sequences between MDA-MB-231 wild type (WT) and HuR CRISPR reveals a precise deletion of 937-956 bp in the HuR sequence, highlighted in yellow.

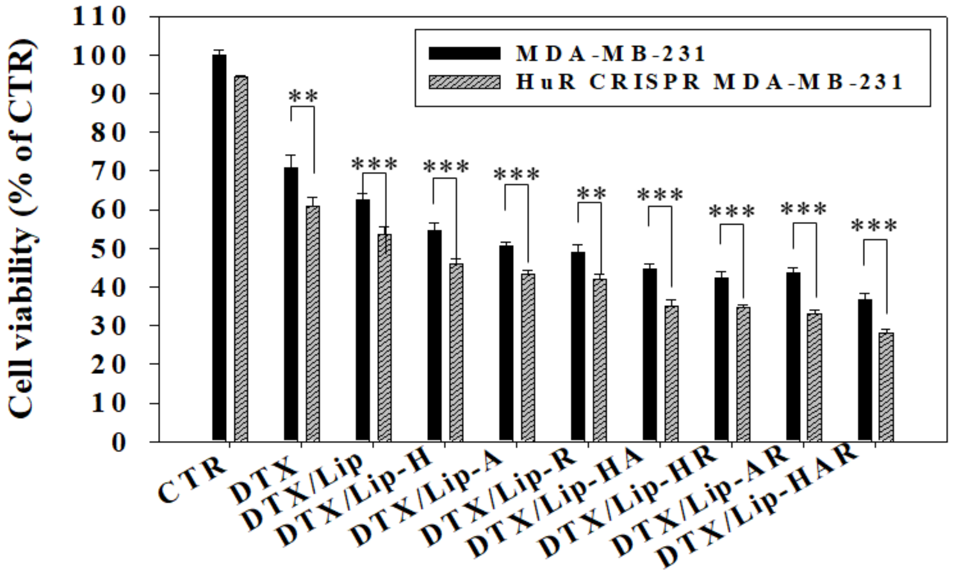

Figure S5. Cytotoxicity of different DTX-loaded formulations. MDA-MB-231 and HuR CRISPR MDA-MB-231 cells were treated with DTX (0.03uM, IC30) for 48 h, and SRB assay was performed (Statistical significance at *$p < 0.05$; **$p < 0.01$; ***$p < 0.001$).

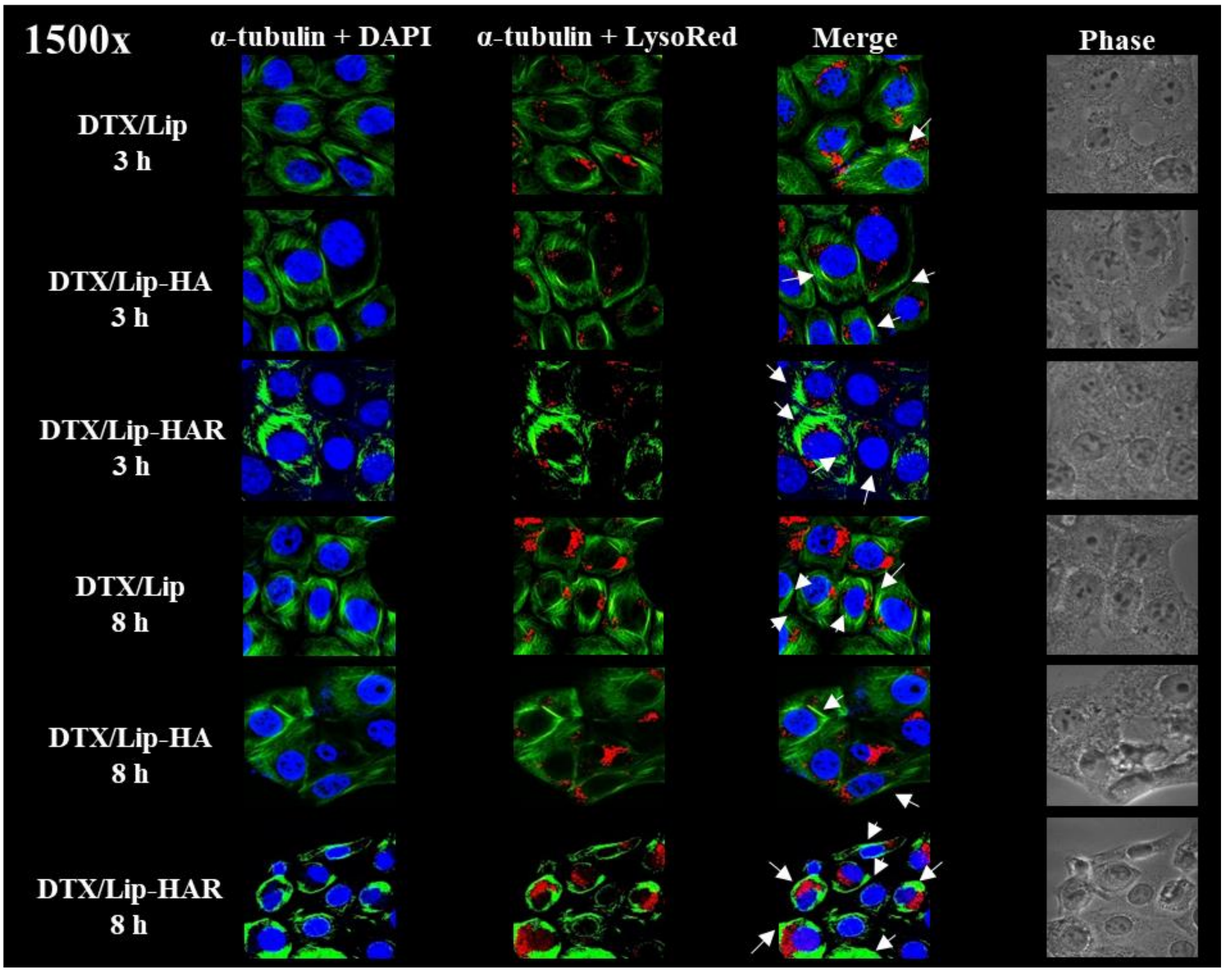

Figure S6. Observation of microtubule fiber alterations in MDA-MB-231 cells following treatment with DTX/Lip, DTX/Lip-HA, and DTX/Lip-HAR at 3 and 8 h. Staining: Blue for DAPI (nuclear dye), green for microtubules, and red for LysoRed (lysosome dye).

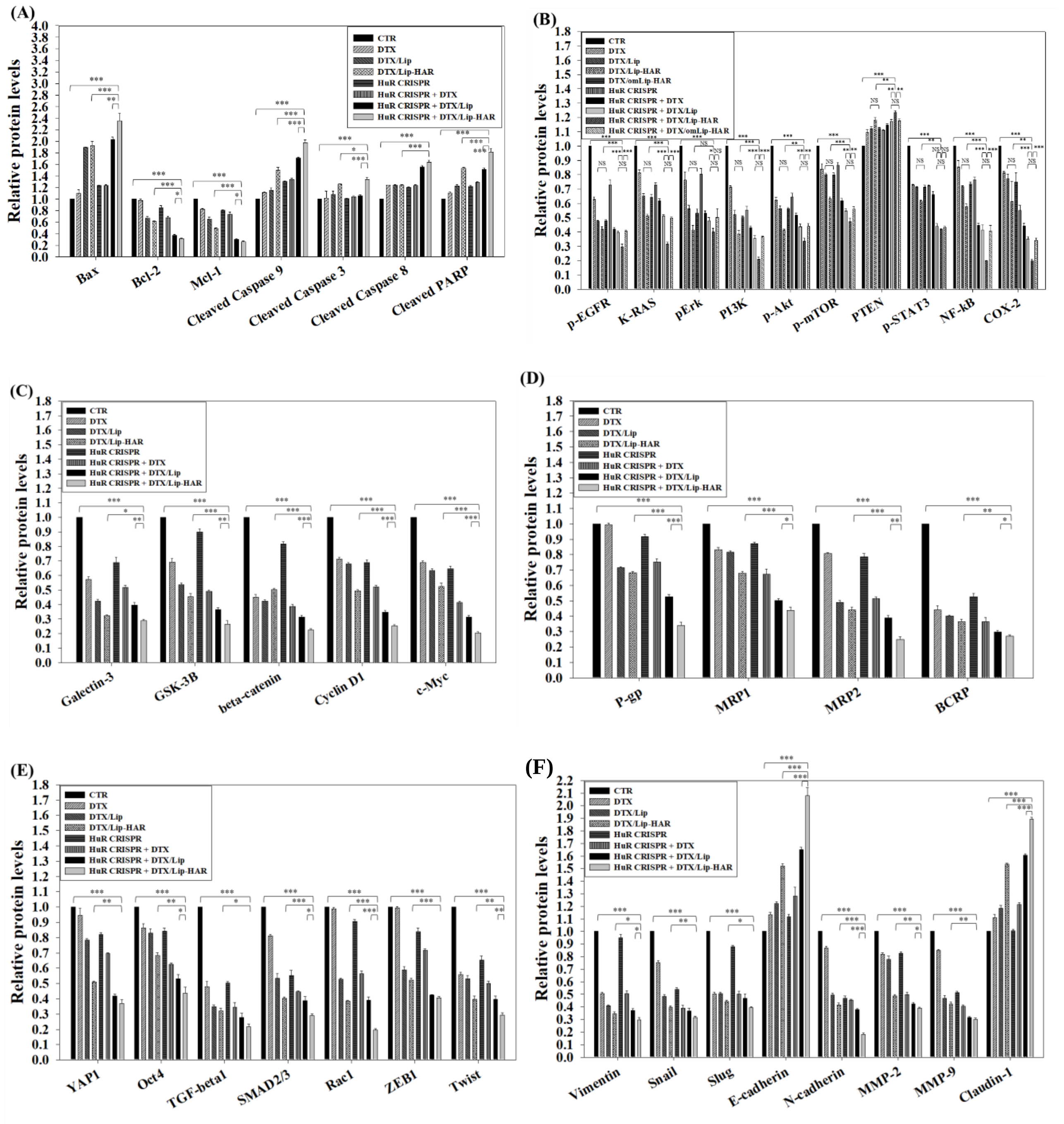

Figure S7. Western blot assay was used to measure the protein levels in the (A) apoptosis-, (B) EGFR-, (C) Wnt-activated, (D) MDR-, and (E-F) EMT-associated pathways in MDA-MB-231 and HuR CRISPR MDA-MB-231 cells administered varied DTX/Lip formulations for 48 h. Protein expression levels in these pathways were assessed using Western blot analysis and quantified using ImageJ (Statistical significance at *$p < 0.05$; **$p < 0.01$; ***$p < 0.001$).

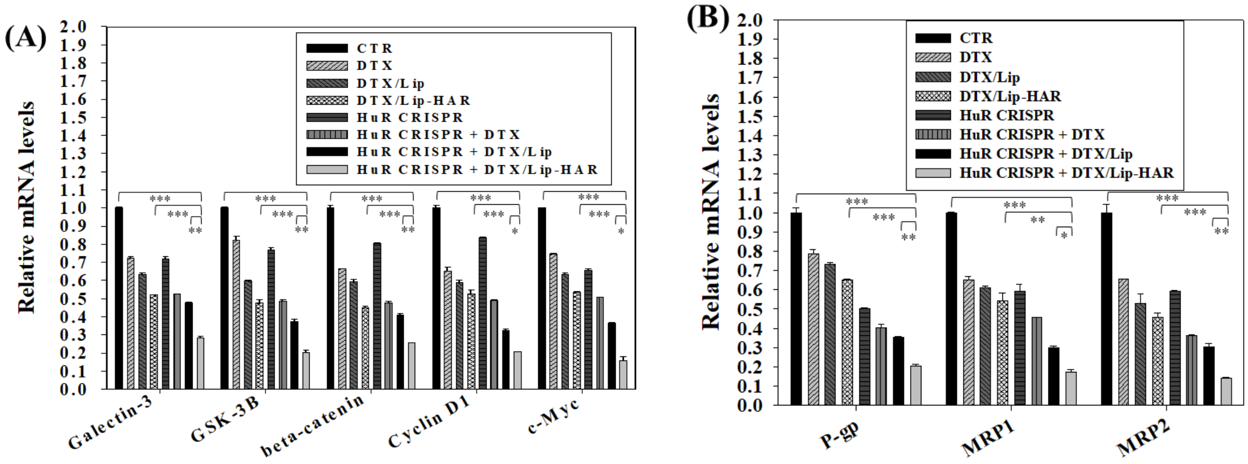

Figure S8. mRNA expression levels of (A) Wnt/β-catenin and (B) MDR-associated pathways in MDA-MB-231 and HuR CRISPR MDA-MB-231 cells were measured by real-time PCR**.** Statistical significance at **p* < 0.05; ***p* < 0.01; ****p* < 0.001.
